# Intrinsic Quantization of Linear Hamiltonian Systems

**DOI:** 10.3390/e28040384

**Published:** 2026-03-31

**Authors:** Luigi Accardi, Carlo Pandiscia

**Affiliations:** Volterra Center, University of Roma Tor Vergata, Via Columbia 2, 00133 Roma, Italy; pandiscia.carlo@gmail.com

**Keywords:** linear Hamiltonian systems, symplectic geometry, geometric quantization, fock representation

## Abstract

This article discusses the quantization of linear Hamiltonian systems, a historically rich but under explored line of research. The key idea is that a classical linear Hamiltonian system induces on its phase space a compatible complex structure and scalar product, giving rise to a complex Hilbert space where classical dynamics becomes a one-parameter unitary group. Boson Fock quantization of this group then recovers, up to unitary equivalence, the results of canonical quantization. This expository overview traces the development of this framework from foundational works to modern symplectic perspectives, offering a case study in the dialogue between analysis, geometry, and physics.

## 1. Introduction

The present paper was born out of a conversation between the first author and Andrei Khrennikov during the the Ernst Strüngmann Forum on *Simplicity behind Absurdity: The Power of Quantum Thinking* (26–31 October 2025, Frankfurt, Germany). In this conversation, Andrei explained his ideas, first discussed in another paper [1], on some pre-quantum structures naturally associated to some classes of classical Hamiltonian models. His ideas turned out to be strictly related to a line of research concerning quantization of linear Hamiltonian systems which, although having a long history with contributions from several known researchers, is not widely discussed in the literature. In the pioneering works on the mathematical foundations of quantum physics, such as those of Segal [2,3], Shale [4], and Araki [5], brought together in a modern synthesis in Folland’s monograph [6], the seeds of this research direction are already present. Among the authors who contributed more specifically to bridging the geometric theory of phase space with quantization, we mention the work of Simms and Woodhouse [7,8], that of Kostant [9], and, from a modern symplectic perspective, the contributions of de Gosson [10]. A first attempt to put together the different approaches to the problem, in the case of linear Hamiltonian systems, was made in a conference of the Italian National Group of Functional Analysis (GNAFA), held in Rimini (October 1977), and published in the proceedings of this conference (see [11]). The basic idea of this approach can be summarized as follows: a classical linear Hamiltonian system (i.e., one with quadratic Hamiltonian) induces on its phase space both a real valued scalar product and a complex structure. The symplectic structure of the classical Hamiltonian system is then used to complexify the original real scalar product in a way compatible with the above-mentioned complex structure. Thus, one obtains a complex Hilbert space in which the classical Hamiltonian evolution becomes a one-parameter unitary group. Finally, applying the boson Fock second quantization procedure to this one-parameter unitary group, one obtains the same result (up to unitary equivalence) to that obtained by applying to the classical Hamiltonian system the ”naive” quantization prescriptions used at the beginning of the theory (a short discussion of it is in Section 2.1).

This line of research, though deeply rooted in the mathematical foundations of quantum theory, can be properly appreciated only when framed within the broader historical and conceptual context that gave rise to quantum mechanics itself.

The attempt to overcome the limitations and contradictions of classical physics that emerged during the nineteenth century and resulted, at the beginning of the twentieth, in two scientific revolutions that fundamentally changed our conception of nature: the theory of relativity and quantum theory.

The first established a completely new foundation for the relationships between space and time, matter, and energy; the second reconciled the age-old dispute between the discrete and continuous viewpoints in describing nature. The attempt to synthesize these two lines of thought led to the discovery of antimatter, which overturned the very concept of the “individuality” of a system, with philosophical and practical consequences certainly comparable to those resulting from the theory of relativity.

Despite these great successes, a complete and satisfactory synthesis of the two theories—that is, the construction of a quantum theory compatible with the requirements of covariance, locality, and causality posed by the theory of relativity (special or general)—still eludes the efforts of the numerous researchers who have devoted themselves, over the last fifty years, to this program.

Since its origins, quantum theory has interacted with the most advanced sectors of mathematical analysis—Lie algebras, representation theory, etc.—and the fruitfulness of this interaction has been such as to fully justify I.M. Gelfand’s statement [12] at the international congress in Amsterdam (1954): *… the study of mathematical problems connected with quantum mechanics was a turning point in the development of functional analysis itself and at the present time, to a great extent, it determines the main paths for its development …*.

The purpose of this paper is to discuss some aspects of this interaction in light of the program mentioned above and from the perspective of a mathematician who seeks in it philosophical inspiration to guide his work, concrete problems to measure and improve his techniques, and stimuli to create new ones. The partiality of this point of view can be overcome only through a deeper interaction with the physical world than current scientific structures seem to allow for.

This work is therefore expository and review-oriented, even if some proofs of known results and some minor statements seem to be new. While the opening discussion sets the stage with the grand, unresolved challenges of relativistic quantum theory, the core of our work will focus on a more circumscribed, yet historically rich and mathematically illuminating, line of inquiry. Our aim is to present this specific thread as a case study, illustrating the profound and fruitful dialogue between physics and mathematics that Gelfand so aptly described, and to offer a mathematician’s perspective on its conceptual underpinnings and enduring relevance.

## 2. Classical and Quantum Description of a System: General Scheme

In non-relativistic classical mechanics, the description of a physical system is achieved by assigning a space *S*—the **states** of the system. The observable quantities associated with the system are represented by a certain set of real functions on *S*. The temporal evolution of the system is described by a two-parameter family of operators,

T(t,s):S→S;s≤t

satisfying

T(s,s)=id;T(t,s)·T(s,r)=T(t,r);r≤s≤t

Such a family will be called an **evolution** on *S*.

The value of any observable *f* at any time *t* is determined by knowing the state *x* of the system at an instant of time s≤t using the following formula:

$$\begin{array}{ccc} f(T(t,s)x) & = & \mathrm{the}\;\mathrm{value}\;\mathrm{of}\;f\;\mathrm{at}\;\mathrm{time}\;t,\;\mathrm{given}\;\mathrm{that}\;\\ & & \mathrm{the}\;\mathrm{state}\;\mathrm{of}\;\mathrm{the}\;\mathrm{system}\;\mathrm{at}\;\mathrm{time}\;s\leq t\;\mathrm{is}\;x \end{array}$$

In general, the space *S* has a structure (measurable, topological, manifold, …), and it is required that the functions f∈A and the operators T(s,t) be compatible with this structure.

In quantum mechanics, the general scheme is the same, in the sense that the fundamental objects of the theory are still a set of observables, a set of states, and an evolution on the state space. What changes radically is the mathematical model of these objects. Indeed, in quantum theory:

(i1) The observables of a physical system are represented by self-adjoint operators on a complex, separable Hilbert space H.
(i2) The states of the system are represented by vectors of norm 1 of H (the two vectors, $\psi_{1},\psi_{2}$, define the same state if and only if $\alpha \psi_{1}=\psi_{2}$ with α∈C, |α|=1). More precisely, these are the “pure” states of the system. In the first part of this exposition, we will consider only states of this type.
(i3) All the statements of the theory can be reduced to expressions of the type (cf. [13], chap. 5))
  $$\begin{array}{ccc} Exp_{\bar{\psi }}\left(\bar{A}\right) & = & \mathrm{Mean}\;(\mathrm{or}\;\mathrm{expected})\;\mathrm{value}\;\mathrm{of}\;\mathrm{the}\;\mathrm{observable}\;\bar{A}\\ & & \mathrm{in}\;\mathrm{the}\;\mathrm{state}\;\bar{\psi }=\langle \psi ,A\psi \rangle \end{array}$$
  where 〈·,·〉 denotes the scalar product in H; $\bar{\psi }$ (resp. $\bar{A}$) is a state (resp. an observable) of the system, and ψ (resp. A) is the corresponding vector of H (resp. self-adjoint operator on H).
  In the following, we will identify states and observables with their mathematical counterparts.

The theory’s prescriptions describe the temporal evolution of a state using a two-parameter family of unitary (and hence is necessarily linear) operators:

$$u\left(t,s\right):H\to H\;,\;s\leq t\;;\;u{\left(t,s\right)}^{*}=u\left(s,t\right)$$

u(s,s)=id;u(t,s)·u(s,r)=u(t,r);r≤s≤t

and therefore the expectation values of an observable in a state at different times:

(1) 〈u(t,s)ψ,Au(t,s)ψ〉:=

expectedvalue,attimet,oftheobservableAifattimes,

thesystemwasinstateψ

**Remark** **1.** It is a known fact that, in classical probability, the definition of conditional probability presupposes that of joint probability. Therefore, the interpretation of the expression $|\langle \psi ,u_{t}^{*}\varphi \rangle {|}^{2}$ as a conditional probability is mathematically justified **from a classical point of view** only if we have a consistent quantum formalism for joint probabilities relative to different instants of time (see [14,15,16]). It is known that, in quantum theory, this is not the case (see [17]).

Another important feature common to the classical and quantum descriptions is the fact that the spatial symmetry properties of the system are defined by the action of particular groups on the state space, and the temporal symmetry properties by the corresponding symmetries in the evolution. For example, the independence of the law of evolution from the initial instant is expressed as

(2) u(s+r,t+r)=u(s,t)

Therefore, u(s,t)=u(t−s) and evolution is a one-parameter group the temporal reversibility of evolution:

(3) $$u\left(s,r\right)=u{\left(r,s\right)}^{-1}\;\left(=\;\mathrm{in}\;\mathrm{the}\;\mathrm{quantum}\;\mathrm{case}\;u{\left(r,s\right)}^{*}\right)$$

Therefore, if (2) and (3) hold, as will be assumed from now on unless explicitly stated otherwise, the evolution is determined by a one-parameter group of automorphisms of the state space: the dynamical group of the system.

The properties listed above, both in the classical and quantum case, define two general schemes for describing physical systems. To move from the general scheme to the actual description of the individual physical system, it is necessary to provide prescriptions that allow us to explicitly construct the system’s state space and its evolution operator starting from the *physical characteristics* of the system itself.

While the general formalism of quantum mechanics (defined by the properties (i1), (i2), and (i3) listed above and the dynamical principle) can be described independently of the corresponding classical formalism, the prescriptions that, to a given physical system, associate its quantum description cannot be assigned independently of the corresponding classical prescriptions.

That is, the quantum description of a given physical system is carried out in two steps:

(1). We consider a classical description of the given physical system;

(2). We move from the classical description to the quantum description.

The requirements that allow us to move from (1) to (2) are called **quantization rules**.

Historically, the formalism of quantum mechanics has developed in close analogy with that of the Hamiltonian description of a system of *n* particles (a detailed description of the historical development of quantum theory is contained in [18]).

### 2.1. Schrodinger Quantization

The Hamiltonian description of a classical isolated system with *n* degrees of freedom and without constraints is carried out, in the phase space $R^{n}\times R^{n}$, by assigning a function

$$H:R^{n}\times R^{n}\to R$$

called the Hamiltonian, which represents the total energy of the system.

The system’s motion occurs on a surface of constant energy

$$H\left(q_{t},p_{t}\right)=E\;;\;\left(q_{t},p_{t}\right)\in R^{n}\times R^{n}\;;\;t\in R$$

and is defined by the evolution law:

(4) $$\frac{d}{dt}f_{t}=\left\{f_{t},H\right\};\;f_{t}=f\left(q_{t},p_{t}\right);\;H_{t}=H$$

where $f:R^{n}\times R^{n}\to R$ is any observable and {·,·} denotes the Poisson brackets:

$$\left\{f,g\right\}=\sum_{j=1}^{n}\left(\frac{\partial f}{\partial q_{j}}\frac{\partial g}{\partial p_{j}}-\frac{\partial f}{\partial p_{j}}\frac{\partial g}{\partial q_{j}}\right)$$

Notice that the Poisson brackets define a **symplectic map**, i.e., it satisfies

{f,g}=−{g,f}

The **Schrodinger quantization** of an Hamiltonian system is performed according to the following requirements:

(i) The quantum state space is $L_{C}^{2}\left(R^{n},dx\right)\equiv L^{2}\left(R^{n}\right)$ where dx the Lebésgue measure.
(ii) Each classical observable $f:R^{n}\times R^{n}\to R$ is associated with the **quantum observable** $\bar{f}$ obtained from the expression
  $$f\left(q,p\right)=f\left(q_{1},\cdots ,q_{n};p_{1},\cdots ,p_{n}\right)$$
  through the formal substitutions:
  $$q_{j}⟼Q_{j}\;;\;\left(Q_{j}f\right)\left(q_{1},\cdots ,q_{n}\right)=q_{j}\cdot f\left(q_{1},\cdots ,q_{n}\right);f\in L^{2}\left(R^{n}\right)$$
  $$p_{j}⟼P_{j}\;;\;P_{j}=\frac{\hbar }{i}\frac{\partial }{\partial q_{j}}$$
(iii) The quantum law of motion is defined by
  (5) $$\frac{d}{dt}A_{t}=-\frac{i}{\hbar }\left[A_{t},\bar{H}\right]\;;\;{\bar{H}}_{t}=\bar{H}$$
  where $\bar{H}=\bar{H}\left(Q,P\right)$ is the operator corresponding to the classical Hamiltonian *H* according to prescription (ii); $A_{t}$ is any quantum observable (self-adjoint operator); [a,b]:=ab−ba denote the commutator between *a* and *b*; and ℏ=h/2π, where *h* is Plank’s constant (cf. [13,19]).

The general solution to Equation (5) is

(6) $$A_{t}=e^{it\bar{H}/\hbar }\cdot A_{0}\cdot e^{-it\bar{H}/\hbar }$$

where $A_{0}$ is the initial data. The duality

(7) $$\langle \psi_{0},A_{t}\psi_{0}\rangle =\langle \psi_{t},A_{0}\psi_{t}\rangle \;;\;\psi_{0},\psi_{t}\in L^{2}\left(R^{n}\right)$$

establishes an equivalence between the evolution (6) of observables and the evolution

(8) $$\psi_{t}=e^{-it\bar{H}/\hbar }\psi_{0}\;;\;\psi_{0}\in L^{2}\left(R^{n}\right)$$

on the state space. The latter, in differential form, is written

(9) $$\frac{d}{dt}\psi_{t}=\frac{1}{i\hbar }\bar{H}\psi_{t}$$

The fact that every strongly continuous unitary evolution has the form (8) for some quantum observable *H* is a fundamental theorem of functional analysis due M. H. Stone [20] (see also J. von Neumann [21]).

#### The Quantization “Algorithm”

The quantization prescriptions formulated in the previous section contain some ambiguities. In fact, the operators $Q_{j}$ and $P_{j}$ are unbounded symmetric operators on $L^{2}\left(R^{n}\right)$ that do not commute with each other. Therefore, the formal correspondence

$$f=f\left(q_{j},p_{j}\right)\to \bar{f}=f\left(Q_{j},P_{j}\right)$$

It is ill-defined for two reasons:

First of all, classical expressions such as

$$q_{j}^{n_{1}}p_{j}^{m_{1}}q_{j}^{n_{2}}p_{j}^{n_{2}}\cdots q_{j}^{n_{k}}p_{j}^{n_{k}}:\;\sum n_{e}=n;\;\sum m_{e}=m$$

coincide with $q^{n}p^{m}$, while this is not true for the corresponding quantum expressions according to rule (ii):

$$Q_{j}^{n_{1}}P_{j}^{m_{1}}\cdots Q_{j}^{n_{k}}P_{j}^{m_{k}}$$

Second, the formal substitutions $q_{j}\to Q_{j};p_{j}\to P_{j}$ in the expression of a classical observable f(q,p) do not always lead to a quantum observable. For example, a polynomial in $q_{j}$ and $p_{j}$, gives rise to an operator *f* that, in general, is not even formally self-adjoint, and therefore does not correspond—not even formally—to a quantum observable. An important class of classical observables in which these formal ambiguities do not arise is given by functions of the type

$$f\left(p,q\right)=g_{1}\left(p\right)+g_{2}\left(q\right)$$

For example, an important family of classical Hamiltonians is of the type

$$H\left(q_{1},\cdots ,q_{n};p_{1},\cdots ,p_{n}\right)=\frac{1}{2m}\sum_{j=1}^{n}p_{j}^{2}+V\left(q_{1},\cdots ,q_{n}\right)$$

where $V:R^{n}\to R$ is a function. In these cases, the corresponding quantum Hamiltonians are well defined (up to domain issues, that we do not discuss here) as symmetric operators on $L^{2}\left(R^{n}\right)$

$$\bar{H}-\frac{{\left(\hbar \right)}^{2}}{2m}\Delta +\bar{V}\left(Q_{1},\cdots ,Q_{n}\right)$$

where

$$\Delta =\frac{\partial^{2}}{\partial q_{1}^{2}}+\cdots +\frac{\partial^{2}}{\partial q_{n}^{2}};\bar{V}\left(Q_{1},\cdots ,Q_{n}\right)=\;\mathrm{operator}\;\mathrm{of}\;\mathrm{multiplication}\;\mathrm{by}\;V$$

However, to associate a quantum observable with these operators (i.e., a self-adjoint operator), it will be necessary to consider self-adjoint extensions of the aforementioned operators, and the possible non-uniqueness of such self-adjoint extensions is the source of another ambiguity in the quantization “algorithm”, as defined by the rules listed in Section 2.1.

The analysis of this type of ambiguities has given rise to a profound line of research on the problem of the essential self-adjointness of the sum of two self-adjoint operators.

This issue will not be discussed here. An extremely lucid exposition and the corresponding bibliography can be found in the lectures of W. Faris [22].

### 2.2. Heisenberg Quantization

The starting point for Heisenberg quantization is the same as for Schrödinger’s: the Hamiltonian description of a system of *n* particles. The requirements are apparently different, namely: Quantizing a system with a classical Hamiltonian $H\left(q,p\right)=H\left(q_{1},\cdots ,q_{n};p_{1},\cdots ,p_{n}\right)$ means establishing a correspondence

(10) $$q_{j}⟼Q_{j}\;\;\;p_{j}⟼P_{j}$$

between the dynamical variables of the system $\left(q_{j},p_{j}\right)$ and 2n self-adjoint operators $Q_{j},P_{j}$ on a separable complex Hilbert space H, such that:

(j) The following commutation rules are satisfied:
  (11) $$\left[Q_{j},P_{k}\right]=\hbar \delta_{k,j}\;(\mathrm{Kroneker}’s\;\mathrm{delta})$$
  (12) $$\left[Q_{j},Q_{k}\right]=\left[P_{j},P_{k}\right]=0$$
(jj) The operator $\bar{H}$ obtained through the formal substitution
  $$H\left(q_{j},p_{j}\right)⟼H\left(Q_{j},P_{j}\right)=\bar{H}$$
  is essentially self-adjoint.

The relations (11), (12) are called **canonical (or Heisenberg) commutation relations**.

A set of self-adjoint operators $Q_{j},P_{j}$ on a Hilbert space H that satisfies (12) is called a representation of the canonical commutation rules.

The operators $Q_{j},P_{j}$ are unbounded operators (cf. Section 2.1), so a precise formulation of the Heisenberg commutation rules requires specifying a subset of the Hilbert space H where they are assumed to hold.

The observation that, on the Hilbert space $L^{2}\left(R^{n}\right)$, the operators

$$Q_{j}=\mathrm{multiplication}\;\mathrm{by}\;\mathrm{the}\;j-\mathrm{th}\;\mathrm{coordinate};$$

$$P_{j}=\left(\hbar /i\right)\partial /\partial q_{j}$$

define *a* representation of the Heisenberg rules is due to Schrödinger [23] and independently to Pauli (in a recently published letter to Jordan [24]). This representation is called the **Schrödinger representation** of the canonical commutation relations.

It is known that, in the formulation (11), (12), the Schrödinger representation of the canonical commutation relations is not unique up to unitary equivalence, even if one adds *irreducibility conditions* (see [25]).

There are formally equivalent formulations of these relations, due to to H. Weyl [19] and later to G. W. Mackey [26,27], which guarantee uniqueness in some technical sense that will not be discussed here.

We can schematically summarize the standard quantization rules as follows:

**(i1.)** $S=R^{n}\times R^{n}\;\mathrm{classical}\;\mathrm{phase}\;\mathrm{space}⟼H\;\mathrm{Hilbert}\;\mathrm{space}\;(\mathrm{any}\;a\;\mathrm{priori}).$

**(i2.)** $q_{j},p_{j}\;\mathrm{fixed}\;\mathrm{coordinates}\;\mathrm{in}\;S⟼Q_{j},P_{j}\;\mathrm{self}-\mathrm{adjoint}\;\mathrm{operators}\;\mathrm{on}\;H$.

They must satisfy the commutation relations (11), (12) on an appropriate domain.

**(i3.)** $f\left(q_{j},p_{j}\right)\;\mathrm{Classical}\;\mathrm{observable}⟼f\left(Q_{j},P_{j}\right)\;\mathrm{Quantum}\;\mathrm{observable}$.

**(i4.)** Classicallawofmotion⟼Quantumlawofmotion:

$$\frac{d}{dt}f_{t}=\left\{f_{t},H\right\}\;⟼\;\frac{d}{dt}A_{t}=-\frac{i}{\hbar }\left[A_{t},H\right]$$

## 3. Linear Classical Hamiltonian Systems

We have seen in the Section 2.1 and Section 2.2 that the Schrödinger–Heisenberg quantization scheme, when applied to a single classical Hamiltonian system, presents various types of ambiguities and, even in cases where such ambiguities do not arise, it limits itself to providing prescriptions for the transition from classical to quantum formalism without clarifying the relationships between the other fundamental objects of the two theories (e.g., states, time evolution, …).

Mackey’s approach, at the cost of assigning a particular role, position, or configuration to an observable, is more satisfactory, not only from a mathematical point of view, but also from a physical one. This, in fact, establishes a precise correspondence between certain symmetries of the classical system (spatial symmetries) and symmetries of the associated quantum system, from which many important features of the quantum formalism (e.g., commutation rules, spin, …) are deduced. But it should be emphasized that all this is limited to the kinematic level: in other words, Mackey’s approach [28] should be considered as a quantization of classical theory as a whole, rather than of the individual dynamical system.

Even Mackey’s further in-depth analysis [27], which aims to classify a priori all quantum Hamiltonians compatible with the Galilean covariance principle, avoids the problem of quantizing the *single, preassigned dynamical system based purely on symmetry considerations*.

In particular, Mackey’s global approach does not clarify the connection between the temporal evolution of a classical system and that of its quantum counterpart.

An important exception to this statement is constituted by **linear Hamiltonian systems**. These describe the small oscillations of a system around a stable equilibrium position (for example, one or more harmonic oscillators, small vibrations of a string or membrane, an electromagnetic field in the absence of charges, …).

Like any Hamiltonian system, the linear ones are characterized by a pair

(S,H)

where S is the state space and *H* is the Hamiltonian. In linear Hamiltonian systems, *H* **is a positive quadratic form on the phase space** S.

It was understood since the early times of quantum theory that, even if S is infinite dimensional, such a system is *equivalent* to a family of one-dimensional harmonic oscillators. However, over the course of many years, the term *equivalent* has acquired a much richer and more precise meaning. The purpose of this section is tho explain in detail this more precise meaning.

We will first discuss the structure of S. The structure of *H* for linear systems will be addressed in Section 4. We limit our discussion to the finite-dimensional case; however, we adopt a general, coordinate-free language to convey the intuition underlying possible infinite dimensional extensions, where further subtleties arise due to topological and domain issues.

However, we have added some short comments on infinite dimensions (see Section 3.1 below) to highlight the subtle problems that arise in this direction.

### 3.1. Algebraic Structure of the Phase Space of a Linear Hamiltonian System

The phase space S of a linear Hamiltonian system is characterized by the following properties.

**Definition** **1.** *Let S be a real vector space on which a **non-degenerate symplectic form** is defined, i.e., a real bi-linear form*

(13) ω:S×S→R

*satisfying*

(14) $$\omega \left(s,s^{\prime }\right)=-\omega \left(s^{\prime },s\right)\;,\;\forall s,s^{\prime }\in S\;\;(\mathrm{Antisymmetry})$$

(15) $$\omega \left(s,s^{\prime }\right)=0\;,\;\forall s^{\prime }\in S\Leftrightarrow s=0\;\;(\mathrm{non}-\mathrm{degenerate})$$

*The pair (S,ω) is called a **symplectic space**.*

*The **canonical transformations** (natural morphisms or symmetries of symplectic spaces) are the linear transformations Λ:S→S that **preserve the symplectic form** (linear symplectic transformations):*

$$\omega \left(\Lambda s,\Lambda s^{\prime }\right)=\omega \left(s,s^{\prime }\right),\;\forall s,s^{\prime }\in S$$

*A canonical transformation* Λ *that also preserves the energy (i.e., the Hamiltonian) is called a **Hamiltonian symmetry**: *

H(Λs)=H(s),∀s∈S

**Remark** **2.** *Degenerate symplectic forms (i.e., not satisfying (15)) naturally appear in quantum central limit theorems (see [29] and references therein), but will not be discussed here.*

#### Topologies Induced by Symplectic Forms

Let (S,ω) be a **symplectic space** in the sense of Definition 1. The symplectic form induces the following duality pairing of S with itself:

(u,v)∈S×S⟼ω(u,v)∈R

Let $\tau_{\omega }$ denote the weak topology on S induced by this pairing, defined by

$$u_{\alpha }\to 0\;\mathrm{if}\;\mathrm{and}\;\mathrm{only}\;\mathrm{if}\;\omega \left(u_{\alpha },v\right)\to 0\;\mathrm{for}\;\mathrm{all}\;v\in S.$$

Thus, the topology $\tau_{\omega }$ is generated by the family of seminorms ${\left\{p_{v}\right\}}_{v\in S}$, where

$$p_{v}\left(u\right)=\left|\omega \left(u,v\right)\right|$$

A topology generated by a family of seminorms makes the space a locally convex topological vector space (LCS).

With this weak topology, S becomes a Hausdorff locally convex topological vector space.

Indeed, the Hausdorff condition is equivalent to the requirement that the family of seminorms separates points. This means that for every u∈S with u≠0, there exists a seminorm $p_{v}$, such that $p_{v}\left(u\right)>0$. In other words, there must exist a v∈S for which |ω(u,v)|>0. This is precisely the non-degeneracy condition of ω,

∀u∈S,u≠0,∃v∈Ssuchthatω(u,v)≠0

which is given by this hypothesis.

Since the family of linear functionals {ω(·,v):v∈S} separates the points of S, the weak topology $\tau_{\omega }$ is Hausdorff.

We denote

(16) $$S^{*}:=\left\{\mathrm{linear}\;\mathrm{functionals}\;\mathrm{on}\;S\;\mathrm{continuous}\;\mathrm{in}\;\mathrm{the}\;\mathrm{topology}\;\tau_{\omega }\right\}$$

(17) $$S^{*,alg}:=\left\{\mathrm{all}\;\mathrm{linear}\;\mathrm{functionals}\;\mathrm{on}\;S\right\}\;\;(\mathrm{algebraic}\;\mathrm{dual}\;\mathrm{of}\;S)$$

In finite dimensional vector spaces, all linear topologies compatible with the vector space structure are equivalent. In particular, every linear functional is automatically continuous. Consequently, the continuity requirement imposes no additional restriction: the topological dual coincides with the algebraic dual.

We recall the following fundamental result concerning weak topologies (see, for instance, [30]):

**Theorem** **1.** Given an element $\varphi \in S^{*}$, there exists a $v_{0}\in S$ such that

(18) $$\varphi \left(u\right)=\omega \left(u,v_{0}\right)\;,\;\forall u\in S$$

This induces the following vector space isomorphism:

(19) $$\omega^{*}:v\in \left(S,\tau_{\omega }\right)\to \omega_{v}^{*}\in {\left(S,\tau_{\omega }\right)}^{*},$$

where, by definition

$$\omega_{v}^{*}\left(u\right):=\omega \left(u,v\right)\;,\;\forall u\in S$$

We know that the map (19) is injective due to the non-degeneracy of the bi-linear form ω. So the essence of Theorem by 1 is its surjectivity, which allows for thew identification:

$$\left(S,\tau_{\omega }\right)∼{\left(S,\tau_{\omega }\right)}^{*}$$

By construction, the isomorphism is weak*-continuous.

### 3.2. Lagrangian Subspaces of Symplectic Spaces

**Definition** **2.** *A non-zero vector subspace M of S is called a **lagrangian subspace** if ω **is identically zero on it**, in this case we write*

(20) $$\omega {|}_{M}=0$$

*and M is maximal with respect to this property.*

**Theorem** **2** (Existence of Lagrangian subspaces). *In any symplectic space (S,ω), lagrangian subspaces exist.*

**Proof.**  If 0≠s∈S, then ω is identically zero on the sub-space R·s≠{0}. Therefore the family of non-zero subspaces of S with this property is not empty. It is clear that, if $M_{\alpha }$ is an increasing chain of non-zero subspaces of S with this property, then ${⋃}_{\alpha }M_{\alpha }$ is still a subspace of S with this property. Therefore by Zorn’s lemma, there exists a non-zero subspace M⊆S maximal with respect to this property. □

**Definition** **3.** *Let M be a vector subspace of S. The* **ω-orthogonal complement of** *M, denoted $M^{{⊥}_{\omega }}$, is defined by*

(21) $$M^{{⊥}_{\omega }}=\left\{u\in V\;,\;\omega (u,l)=0\;,\;\forall l\in M\right\}$$

**Remark** **3.** *Notice that M is a lagrangian subspace of S iff*

(22) $$M=M^{{⊥}_{\omega }}$$

*In fact (22) is equivalent to say that ω is identically zero on L and maximal with respect to this property and this is the definition of lagrangian subspace.*

**Lemma** **1.** *If $M_{1}$ and $M_{2}$ are arbitrary vector subspaces of S, then*

(23) $${\left(M_{1}+M_{2}\right)}^{{⊥}_{\omega }}={\left(M_{1}\right)}^{{⊥}_{\omega }}\cap {\left(M_{2}\right)}^{{⊥}_{\omega }}$$

**Proof.**  It is clear that

$${\left(M_{1}\right)}^{{⊥}_{\omega }}\cap {\left(M_{2}\right)}^{{⊥}_{\omega }}\subseteq {\left(M_{1}+M_{2}\right)}^{{⊥}_{\omega }}$$

The converse inclusion follows because, if $s\in {\left(M_{1}+M_{2}\right)}^{{⊥}_{\omega }}$, then in particular $s\in {\left(M_{1}\right)}^{{⊥}_{\omega }}$ and $s\in {\left(M_{2}\right)}^{{⊥}_{\omega }}$, i.e., $s\in {\left(M_{1}\right)}^{{⊥}_{\omega }}\cap {\left(M_{2}\right)}^{{⊥}_{\omega }}$. □

### 3.3. The Natural Embeddings

Let M be a subspace of S. We define the following map:

(24) $$\hat{\omega }:S\to M^{*}\;,\;m\in S\to \hat{\omega }\left(m\right):=\omega \left(m,\cdot \right)\in M^{*}$$

In this case, we have

$$\ker\hat{\omega }=M^{{⊥}_{\omega }}$$

The map $\hat{\omega }$ is surjective. Indeed, by the Hahn–Banach theorem, for every $\varphi_{o}\in M^{*}$, one can extend it to a $\varphi \in S^{*}$. Since the symplectic form ω induces an isomorphism between S and its continuous dual $S^{*}$, there exists a $v_{o}\in S$, such that $\varphi \left(u\right)=\omega \left(u,v_{o}\right)$ for every u∈S, and thus in particular for every m∈M. This proves the surjectivity of $\hat{\omega }$, i.e., Imω^=M*.

Since $\hat{\omega }$ is surjective with kernel $M^{{⊥}_{\omega }}$, it descends to an *algebraic isomorphism* (as vector spaces):

(25) $$S/M^{{⊥}_{\omega }}≃M^{*}$$

By the universal property of the quotient topology (see [31]), the isomorphism in (25) *is topological* if and only if the map $\hat{\omega }$ defined in (24) is open, a condition that is not always satisfied in the infinite-dimensional setting.

In **finite dimensions**, the situation is simpler. Since every linear map on a finite-dimensional space is continuous, the map $\hat{\omega }$ is automatically continuous. Moreover, by a standard algebraic fact, the surjectivity of $\hat{\omega }$ yields the internal direct sum decomposition

S=kerω^+˙Imω^

which gives the isomorphism

$$S≃M^{{⊥}_{\omega }}⊕M^{*}.$$

If, in addition, M is Lagrangian, then $M^{{⊥}_{\omega }}=M$, and we obtain the decomposition

(26) $$S≃M⊕M^{*}$$

Consequently, in finite dimension, (25) and (26) are *topological isomorphisms*. Note also that

$$\dim S=\dim M^{{⊥}_{\omega }}+\dim M$$

and since $\dim M=\dim M^{*}$, it follows that, if M is Lagrangian, we obtain

dimS=2dimM

### 3.4. Existence of a Lagrangian Decomposition

**Definition** **4.** *A* Lagrangian decomposition *of a symplectic space (S,ω) is a decomposition of S as the direct sum of two Lagrangian subspaces.*

Formally, a pair of subspaces $M_{+},M_{-}\subset S$ is a Lagrangian decomposition if

I. Both $M_{+}$ and $M_{-}$ are Lagrangian;
II. They intersect trivially, i.e., $M_{+}\cap M_{-}=\left\{0\right\}$;
III. The space S can be written as the direct sum
  $$S=M_{+}\dot{+}M_{-}$$
  This means that every element v∈S can be uniquely written as $v=v_{+}+v_{-}$ with $v_{+}\in M_{+}$ and $v_{-}\in M_{-}$.

**Theorem** **3.** *Let M be a Lagrangian subspace of S and define the family*

(27) $$F_{M}:=\left\{S_{0}\subseteq_{sub-sp.}S\;:\;S_{0}\subset S_{0}^{{⊥}_{\omega }}\mathrm{and}S_{0}\cap M=\left\{0\right\}\right\}$$

*Then*

*(i)*  *$F_{M}$ is non-empty and contains a maximal element different from {0}.*
*(ii)*  *Let $M^{\prime }$ be a maximal element in $F_{M}$. Then*
(28) $$M\cap M^{\prime }=\left\{0\right\}$$
  *and*
$$M^{\prime }=M^{\prime {⊥}_{\omega }}\Leftrightarrow M^{\prime }\dot{+}M=S$$
*(iii)*  *If S is **finite-dimensional**, then*
(29) $$M\dot{+}M^{\prime }=S$$

**Proof.**  (i) $F_{M}\neq \emptyset$.

Since it is always possible to find a nonzero element $s_{o}\in S$ such that $\omega (s_{o},s_{o})=0$, we can consider the space $S_{o}=R\cdot s_{o}$, clearly $S_{o}\in F_{M}$.

Let $\left(S_{\alpha }\right)$ be an increasing family in $F_{M}$. Then

$$S_{1}:=\underset{\alpha }{⋃}S_{\alpha }$$

is a vector subspace of S such that, if $u,v\in S_{1}$, our assumption implies that there exists β such that $u,v\in S_{\beta }$, hence

ω(u,v)=0

because $S_{\beta }\in F_{M}$. So $S_{1}$ satisfies condition (i) in (27). Furthermore, if $0\neq w\in S_{1}$, then, for some β, $w\in S_{\beta }$; hence, there exists $m_{w}\in M$, such that $\omega (w,m_{w})\neq 0$. So $S_{1}$ also satisfies condition (ii) in (27). Therefore, $S_{1}\in F_{M}$ and by Zorn’s lemma $F_{M}$ contains a maximal element. This proves (i).

From the lemma (1), we have the following relation:

$$M^{\prime {⊥}_{\omega }}\dot{+}M^{{⊥}_{\omega }}={\left(M^{\prime }\dot{+}M\right)}^{{⊥}_{\omega }}$$

hence,

$$M^{\prime {⊥}_{\omega }}\dot{+}M={\left(M^{\prime }\dot{+}M\right)}^{{⊥}_{\omega }}$$

if

$$M^{\prime }\dot{+}M=S⟹M^{\prime {⊥}_{\omega }}\dot{+}M=0$$

thus, $M^{\prime {⊥}_{\omega }}\in F_{M}$ and by maximality $M^{\prime }=M^{\prime {⊥}_{\omega }}$. The converse is trivial. This proves (ii). For (iii), we have the following.

Let dimS=2n. Since M is Lagrangian, there exists a symplectic (or Darboux) basis $\left\{e_{1},\ldots ,e_{n},f_{1},\ldots ,f_{n}\right\}$ of S such that

- $\left\{e_{1},\ldots ,e_{n}\right\}$ is a basis of M;
- $\omega (e_{i},e_{j})=0$, $\omega (f_{i},f_{j})=0$, $\omega \left(e_{i},f_{j}\right)=\delta_{ij}$ for all i,j=1,…,n.

Consider the subspace

$$V=\mathrm{Span}\{f_{1},\ldots ,f_{n}\}.$$

By construction, V is Lagrangian ($V=V^{{⊥}_{\omega }}$) and V∩M={0}. In particular, $V\subset V^{{⊥}_{\omega }}$, so $V\in F_{M}$. We have

$$M^{\prime }\dot{+}M\subset S=V\dot{+}M⟹M^{\prime }\subset V,$$

hence, by maximality, $M^{\prime }=V$. □

**Theorem** **4.** *In finite dimensions case, we have the following identifications:*

(30) $$S∼M⊕M^{*}∼M\dot{+}M∼M\dot{+}M^{\prime }$$

*In particular,*

(31) $$M^{\prime }∼M^{*}∼M$$

**Proof.**  By applying the preceding results, one simply observes that, if dimS=2n, then $\dim M^{*}=\dim M=n$; moreover, since $M^{\prime }$ is Lagrangian, it follows that $\dim M^{\prime }=n$ as well. □

**Remark** **4.** ***Case of Topological Vector Spaces or Hilbert spaces**.*

*In many papers, the space S is endowed with a topology τ that is independent of the symplectic form ω. In such cases, ω is usually required to be continuous with respect to τ. However, in infinite dimensions, the same topology on S may be compatible with several different topologies on the space of bilinear forms. For instance, if S is a Hilbert space, a bilinear form may be norm-continuous, weakly continuous, strongly continuous, and so on. This illustrates how subtle distinctions arise as soon as topologies are introduced in infinite dimensional settings.*

In particular, we must distinguish between the dual ${\left(S,\tau_{\omega }\right)}^{*}$ and the dual ${\left(S,\tau \right)}^{*}$. Since the continuity of ω implies that the topology $\tau_{\omega }$ induced by ω is weaker than τ, that is, $\tau_{\omega }\subset \tau$, we immediately obtain the inclusion

$${\left(S,\tau \right)}^{*}\subset S^{*}:={\left(S,\tau_{\omega }\right)}^{*}$$

*This observation is crucial for distinguishing between **weak** and **strong** non-degeneracy. In fact, a symplectic form ω is said to be* weakly non-degenerate *when only condition (II) of non-degeneracy is satisfied, whereas it is* strongly non-degenerate *when the associated map (19)*

(32) $$\omega^{♯}:\left(S,\tau \right)⟶{\left(S,\tau \right)}^{*},\;v\mapsto \omega \left(v,\cdot \right)$$

is a topological isomorphism onto the whole dual ${\left(S,\tau \right)}^{*}$.

*The motivation for introducing this distinction is provided by the relation in (25), which plays a decisive role in ensuring the existence of a Lagrangian decomposition.*

### 3.5. The Canonical Form of a Symplectic Space

In this section, we use the identities (30) of Theorem (4) in the form

(33) $$S∼M\dot{+}M^{\prime }∼M\dot{+}M^{*}∼M\dot{+}M\;;\;M∼M^{\prime }∼M^{*}$$

and we denote

(34) $$m^{\prime }\in M^{\prime }\mapsto j_{m^{\prime }}^{*}\in M^{*}$$

the isomorphism characterized by

(35) $$j_{m^{\prime }}^{*}\left(m\right):=\omega \left(m^{\prime },m\right)\;,\;m^{\prime }\in M^{\prime }\;,\;m\in M$$

**Theorem** **5.** *In the above notations, for any $m_{1},m_{2}\in M$ and $m_{1}^{\prime },m_{2}^{\prime }\in M^{\prime }$, one has,*

(36) $$\omega \left(m_{1}+m_{1}^{\prime },m_{2}+m_{2}^{\prime }\right)=j_{m_{1}^{\prime }}^{*}\left(m_{2}\right)-j_{m_{2}^{\prime }}^{*}\left(m_{1}\right)$$

**Proof.**  Using the fact that ω is identically zero on both M and $M^{\prime }$, one finds

$$\omega \left(m_{1}+m_{1}^{\prime },m_{2}+m_{2}^{\prime }\right)=\omega \left(m_{1},m_{2}\right)+\omega \left(m_{1},m_{2}^{\prime }\right)+\omega \left(m_{1}^{\prime },m_{2}\right)+\omega \left(m_{1}^{\prime },m_{2}\right)$$

$$=\omega \left(m_{1},m_{2}^{\prime }\right)+\omega \left(m_{1}^{\prime },m_{2}\right)=\omega \left(m_{1}^{\prime },m_{2}\right)-\omega \left(m_{2}^{\prime },m_{1}\right)$$

$$\overset{\left(35\right)}{=}j_{m_{1}^{\prime }}^{*}\left(m_{2}\right)-j_{m_{2}^{\prime }}^{*}\left(m_{1}\right)$$

which is (36). □

**Remark** **5.** *Using the identifications (33) in the form*

S∼M+˙M′∼M+˙M*≡qp:q∈M,p∈M*

and denoting ${\langle \;\cdot \;\;\cdot \;\rangle }_{M^{*},M}$ the duality $\langle M^{*}\;,\;M\rangle$, we can write (36) in the more familiar form

(37) $$\omega \left(q_{1}+p_{1},q_{2}+p_{2}\right)={\langle p_{1},q_{2}\rangle }_{M^{*},M}-{\langle p_{2},q_{1}\rangle }_{M^{*},M}$$

**Remark** **6.** *In the following, we will freely use the identifications (33).*

## 4. Hamiltonian Systems: Dynamics

In this section, we briefly recall some standard facts about Hamiltonian systems with linear phase space. To make the connection with the notations introduced in the previous sections, we introduce the identifications

$$M^{*}\equiv {\left(R^{n}\right)}^{*}\equiv R^{n}\equiv M$$

and we fix the identification (see (33))

$$S:=R^{n}\dot{+}R^{n}\equiv M\dot{+}M$$

Starting from Newton’s equation

(38) $$F=ma\Leftrightarrow F\left(x\right)=m\ddot{x}=\frac{d}{dt}\left(m\dot{x}\right)\;;\;x\in M$$

and defining the momentum and position coordinates by

$$m\dot{x}=:p\in M\;;\;x=:q\Leftrightarrow \dot{q}=\frac{1}{m}p\;,\;q\left(0\right)=q_{0}$$

Newton’s equation (38) becomes equivalent to the system

(39) $$\dot{q}=\frac{1}{m}p\;,\;\dot{p}=F\left(x\right)\;q\left(0\right)=q_{0}\;,\;p\left(0\right)=p_{0}$$

The equations of motion (39) can be written in the equivalent form:

(40) $$\frac{d}{dt}\left(\begin{array}{c} q\\ p \end{array}\right)=\left(\begin{array}{c} p/m\\ F(q) \end{array}\right)$$

### 4.1. Hamiltonian Formalism

Introducing the Hamiltonian

(41) H(q,p)=12mp2+V(q);qp∈M+˙M

Newton’s equation (39) becomes equivalent to the system

(42) $$\dot{q}=\frac{\partial H}{\partial p}=\partial_{p}H=\frac{p}{m}\;;\;\dot{p}=-\frac{\partial H}{\partial q}=-\partial_{q}H=-\partial_{q}V\left(q\right)$$

which can be written in unified form

(43) $$\frac{d}{dt}\left(\begin{array}{c} q\\ p \end{array}\right)=\left(\begin{array}{cc} 0 & 1\\ -1 & 0 \end{array}\right)\left(\begin{array}{c} \partial_{q}H\left(q,p\right)\\ \partial_{p}H\left(q,p\right) \end{array}\right)=:J\nabla H\left(q,p\right)$$

where

$$J:=\left(\begin{array}{cc} 0 & 1\\ -1 & 0 \end{array}\right)$$

### 4.2. The Case of Quadratic Hamiltonians (Linear Hamiltonian Systems)

By definition, a quadratic Hamiltonian has the form

(44) $$H\left(q,p\right)=\bar{T}\left(p,p\right)+\bar{V}\left(q,q\right)\;,\;\left(\begin{array}{c} q\\ p \end{array}\right)\in M\dot{+}M$$

and both $\bar{T}\left(\;\cdot \;,\;\cdot \;\right)$ and $\bar{V}\left(\;\cdot \;,\;\cdot \;\right)$ are bi-linear forms on M:

$$\bar{T}\left(\;\cdot \;,\;\cdot \;\right)\;,\;\bar{V}\left(\;\cdot \;,\;\cdot \;\right):{\left(M\dot{+}M\right)}^{2}\to R$$

We assume that both bi-linear forms (or equivalently *H*) are strictly positive-definite. Bi-linearity implies that there exist linear maps

$$T,V:M\to M^{*}\equiv M$$

such that

(45) $$\partial_{q}H\left(q,p\right)=Vq\;;\;\partial_{p}H\left(q,p\right)=Tp\;,\;\forall q,p\in M$$

#### The Equations of Motion

Equation (45) implies that the equations of motion (43) take the form

$$\frac{d}{dt}\left(\begin{array}{c} q\\ p \end{array}\right)=\left(\begin{array}{cc} 0 & 1\\ -1 & 0 \end{array}\right)\left(\begin{array}{c} \partial_{q}H\left(q,p\right)\\ \partial_{p}H\left(q,p\right) \end{array}\right)=J\left(\begin{array}{c} Vq\\ Tp \end{array}\right)$$

(46) $$=\left(\begin{array}{cc} 0 & 1\\ -1 & 0 \end{array}\right)\left(\begin{array}{cc} V & 0\\ 0 & T \end{array}\right)\left(\begin{array}{c} q\\ p \end{array}\right)=\left(\begin{array}{cc} 0 & T\\ -V & 0 \end{array}\right)\left(\begin{array}{c} q\\ p \end{array}\right)$$

In order to simplify calculations it is convenient to introduce the **kinetic energy scalar product**:

(47) $$\bar{T}\left(p,p\right)=:\frac{1}{2}{\langle p,p\rangle }_{T}$$

We further suppose that, for some hermitian linear Hilbert space operator,

$$F:\left(M,{\langle \;\cdot \;,\;\cdot \;\rangle }_{T}\right)\to \left(M,{\langle \;\cdot \;,\;\cdot \;\rangle }_{T}\right)$$

one has

(48) $$\bar{V}\left(q,q\right)=\frac{1}{2}{\langle q,Fq\rangle }_{T}$$

Notice that *F* is injective (hence invertible in finite dimensions) because the quadratic form $\bar{V}$ is strictly positive.

With this choice, we are privileging momentum over position; however, due to our assumptions, the two can be treated symmetrically.

Given (48), the quadratic Hamiltonian (44) is implemented by the symmetric positive definite bi-linear form on $M\dot{+}M$:

(49) H((q,p),(q′,p′))=12〈p,p′〉T+12〈q,Fq′〉T=:qp,q′p′H

So, defining

(50) qp,q′p′T+˙T:=12〈p,p′〉T+12〈q,q′〉T

we obtain the scalar product

(51) qp,q′p′H=qp,F001q′p′T+˙T

Note that, with our identifications, the scalar products (50) and (51) **act on the same vector space** $M\dot{+}M$.

The solution of the equations of motion in this case is discussed in Appendix A.

### 4.3. The Hamiltonian Evolution as a One-Parameter Group of Rotations on the Real Hilbert Space $\left(M\dot{+}M,{\langle \;\cdot \;,\;\cdot \;\rangle }_{H}\right)$

The perspective on Hamiltonian evolution as a one-parameter group of rotations on a real Hilbert space is closely related to the geometric formulations of quantum mechanics developed by Kibble [32], Ashtekar and Schilling [33], and later by Brody and Hughston [34], as well as to the symplectic framework introduced earlier by Souriau [35,36]. All these approaches emphasize the Hamiltonian and symplectic nature of quantum evolution and are naturally connected to the pre-quantum frameworks discussed above.

Conservation of energy for Hamiltonian systems implies that the Hamiltonian evolution

(52) $$V_{t}=e^{tA}\;,\;t\in R$$

is a one-parameter group of orthogonal transformations in the real Hilbert space $M\dot{+}M$ **with scalar product (49) defined by the Hamiltonian**, i.e.,

(53) H((q,p),(q′,p′))=12〈p,p′〉T+12〈q,Fq′〉T=:qp,q′p′H

We call this the *H*–**scalar product**. Computations will be simpler in the **kinetic energy scalar product** (50) acting on the same vector space, i.e.,

(54) qp,q′p′T+˙T:=12〈p,p′〉T+12〈q,q′〉T

we call it simply the **T+T scalar product**. Recall that the relation between the two is

(55) qp,q′p′H=qp,F001q′p′T+˙T

Since the generator of a one-parameter group of orthogonal transformations in a real Hilbert space is a **skew adjoint operator**, this naturally leads us to compute the polar decompositions of the generator of the Hamiltonian evolution in the *H*–scalar product. We will see in Section 4.4 that the complex structure on the classical (real) phase space naturally arises from these polar decompositions.

#### The Polar Decompositions of the Operator *A* in the *H*– and in the T+T–Scalar Products

We want to calculate the polar decomposition of the operator *A* defined by (A2), i.e.,

(56) $$A:=\left(\begin{array}{cc} 0 & 1\\ -F & 0 \end{array}\right)$$

with respect to the *H*–scalar product (49) on $M\dot{+}M$, i.e.,

(57) H(q,p)=12〈p,p〉T+12〈q,Fq〉T=qp,qpH

=()qp,F001q′p′T+˙T

For any linear operator *X* on $M\dot{+}M$, we denote

(58) $$\left\{\begin{array}{cc} X^{*}, & \mathrm{the}\;T\dot{+}T-\mathrm{adjoint}\;\mathrm{of}\;X\\ X^{+}, & \mathrm{the}\;H-\mathrm{adjoint}\;\mathrm{of}\;X \end{array}\right.$$

**Proposition** **1.** (i) In the notation (58), $X^{*}$ and $X^{+}$ are related by the identity

(59) $$X^{+}=\left(\begin{array}{cc} F^{-1} & 0\\ 0 & 1 \end{array}\right)X^{*}\left(\begin{array}{cc} F & 0\\ 0 & 1 \end{array}\right)$$

*(ii) Denoting ${\left|A\right|}_{T}$ the module of A in the $\left(T\dot{+}T\right)$–scalar product, one has*

(60) $${\left|A\right|}_{T}=\sqrt{A^{*}A}=\left(\begin{array}{cc} F & 0\\ 0 & 1 \end{array}\right)$$

*(iii) Denoting*

(61) $$J_{T}=\left(\begin{array}{cc} 0 & 1\\ -1 & 0 \end{array}\right)$$

*the polar decomposition of A in the $\left(T\dot{+}T\right)$–space is given by*

(62) $$A=J_{T}{\left|A\right|}_{T}=\left(\begin{array}{cc} 0 & 1\\ -1 & 0 \end{array}\right)\left(\begin{array}{cc} F & 0\\ 0 & 1 \end{array}\right)$$

*(iv) The H–adjoint of A is (as we already know from the remark at the end of Section 4.3)*

(63) $$A^{+}=-A$$

*(v) Denoting ${\left|A\right|}_{H}$ the module of A in the H–scalar product, one has*

(64) $${\left|A\right|}_{H}=\sqrt{A^{+}A}=\left(\begin{array}{cc} F^{1/2} & 0\\ 0 & F^{1/2} \end{array}\right)$$

*(vi) Denoting*

(65) $$J_{H}:=\left(\begin{array}{cc} 0 & F^{-1/2}\\ -F^{1/2} & 0 \end{array}\right)$$

*the polar decomposition of A in the H–space is given by (see (56))*

(66) $$A=J_{H}{\left|A\right|}_{H}=\left(\begin{array}{cc} 0 & F^{-1/2}\\ -F^{1/2} & 0 \end{array}\right)\left(\begin{array}{cc} F^{1/2} & 0\\ 0 & F^{1/2} \end{array}\right)$$

*(vii) The H–adjoint of $J_{H}$ is given by*

(67) $$J_{H}^{+}=-J_{H}$$

*Moreover $J_{H}$ satisfies*

(68) $$J_{H}^{2}=-1$$

The proof of this statement is in Appendix B.

### 4.4. Canonical H-Dependent Complex Structure on $M\dot{+}M$

Let us explain the main idea of this section. We start from the polar decomposition of the Hamiltonian evolution on $M\dot{+}M$,

(69) $$e^{tA}=e^{tJ_{H}{\left|A\right|}_{H}}\;,\;t\in R$$

where $J_{H}$ satisfies

(70) $$J_{H}^{+}\overset{\left(67\right)}{=}-J_{H}\;;\;J_{H}^{2}\overset{\left(68\right)}{=}-1$$

We use these properties to define a complex structure on $M\dot{+}M$ in which $J_{H}$ acts as **imaginary unit**.

Moreover, ${\left|A\right|}_{H}$ is a positive operator in the *H*–space satisfying

(71) $$A=J_{H}{\left|A\right|}_{H}=\left(\begin{array}{cc} 0 & F^{-1/2}\\ -F^{1/2} & 0 \end{array}\right)\left(\begin{array}{cc} F^{1/2} & 0\\ 0 & F^{1/2} \end{array}\right)=\left(\begin{array}{cc} 0 & 1\\ -F & 0 \end{array}\right)$$

$$=\left(\begin{array}{cc} F^{1/2} & 0\\ 0 & F^{1/2} \end{array}\right)\left(\begin{array}{cc} 0 & F^{-1/2}\\ -F^{1/2} & 0 \end{array}\right)={\left|A\right|}_{H}J_{H}$$

i.e., ${\left|A\right|}_{H}$ commutes with $J_{H}$. In terms of the above-mentioned complex structure, this means that ${\left|A\right|}_{H}$ is a **complex linear operator**.

Then we define a new, complex valued scalar product on the new complex vector space (isomorphic, as a real vector space, to $M\dot{+}M$), compatible with with the complex structure and such that positive operator ${\left|A\right|}_{H}$ in the *H*–space is still positive with respect to the complex scalar product.

In this new scalar product, the right-hand side of (69), i.e., $\left(e^{tJ_{H}{\left|A\right|}_{H}}\right)$, can be interpreted as a one-parameter **unitary** group with positive generator ${\left|A\right|}_{H}$. In conclusion:

*Any linear Hamiltonian evolution on a (necessarily linear) real phase space naturally defines a complex Hilbert space structure on the phase space with respect to which the initial evolution becomes unitary.*

In the following, we discuss in more detail the construction outlined above.

#### 4.4.1. The Identification $M\dot{+}M\equiv M\dot{+}J_{H}M$

Note that, for each q∈M,

(72) JHq0=0F−1/2−F1/20q0=0−F1/2q

and, since $-F^{1/2}$ is invertible, (72) implies

(73) JHM0=0M

Moreover,

JH0−F1/2q=JH2q0=(70)−q0

so, in the identifications

M+˙M≡(M+˙{0})+˙({0}+˙M)≡M0+˙0M

**Lemma** **2.** *The map*

(74) $$u:z=a+ib\in C\to u\left(z\right):=a+J_{H}b\in \;\mathrm{linear}\;\mathrm{operators}\;\mathrm{on}\;S$$

is a isomorphism of *–algebras (in fact, since $J_{H}$ is bounded on the real H–Hilbert space $M\dot{+}M$, of $C^{*}$–algebras [37]).

**Proof.**  Additivity is clear. The *–map property follow from

$$u\left(\bar{z}\right)=u\left(\bar{a+ib}\right)=u\left(a+i\left(-b\right)\right)=a+J_{H}\left(-b\right)=a-J_{H}b={\left(a+J_{H}b\right)}^{+}=u{\left(z\right)}^{+}$$

given the above, the multiplicativity is equivalent to $J_{H}^{2}=-1$ which follows from (70). □

The above considerations define a complex structure on S as follows.

Define

(75) SR:=M0≡M+˙{0}

and consider the vectors space direct sum decomposition

(76) SC=SR+˙JHSR=q0+JHq′0:q,q′∈M

=(72)q−F1/2q′:q,q′∈M

The complex linear operators on $S_{C}$ are those real linear operators on S, which commute with the complex structure. This is equivalent to say that they commute with $J_{H}$. The following result will not be used, but we include it for completeness.

**Theorem** **6.** *The complex–linear operators on $S_{C}$ are the operators of the form*

$$\left(\begin{array}{cc} B & C\\ -F^{1/2}CF^{1/2} & F^{1/2}BF^{-1/2} \end{array}\right)$$

*where B and C are arbitrary real linear operators on $S_{R}$.*

The proof of this statement is in Appendix B.

#### 4.4.2. The Complex Valued Scalar Product on $M\dot{+}J_{H}M$

**Theorem** **7.** *On the real Hilbert space*

$$S=M\dot{+}J_{H}M\equiv M\dot{+}M$$

*with real valued H–scalar product ${\langle \;\cdot \;|\;\cdot \;\rangle }_{H}$ and with the real linear involution $J_{H}$, the real bi–linear form 〈·,·〉 on S, defined by*

$$\langle m_{1}+J_{H}m_{2},m_{1}^{\prime }+J_{H}m_{2}^{\prime }\rangle :=$$

(77) $$:={\langle m_{1},m_{1}^{\prime }\rangle }_{H}+{\langle m_{2},m_{2}^{\prime }\rangle }_{H}+i\left({\langle m_{1},m_{2}^{\prime }\rangle }_{H}-{(m_{2},m_{1}^{\prime }\rangle }_{H}\right)$$

*($m_{1},m_{2},m_{1}^{\prime },m_{2}^{\prime }\in M$) is in fact a complex valued pre–scalar product on $S_{C}$, i.e., S considered as a complex vector space with the action of C defined by (74). The pre–scalar product 〈·,·〉 is compatible with the complex structure induced by $J_{H}$ in the sense that:*

*(i) The restriction of the pre–scalar product on M is real valued.*

*(ii) One has*

(78) $$\langle m,J_{H}m^{\prime }\rangle =i{\langle m,m^{\prime }\rangle }_{H}\in iR\;;\;\forall m,m^{\prime }\in M$$

*(iii) The operator ${\left|A\right|}_{H}$ is positive in the complex Hilbert space $\left(S_{C},\langle \;\cdot \;,\;\cdot \;\rangle \right)$.*

*(iv) The one-parameter group of orthogonal transformation of M*

(79) $$V_{t}:=e^{tJ_{H}{\left|A\right|}_{H}}\equiv e^{itH}\;,\;t\in R$$

*is unitary in the Hilbert space $\left(S_{C},\langle \;\cdot \;,\;\cdot \;\rangle \right)$.*

**Proof.**  That the right-hand side of (77) is a pre–scalar product on M is clear because, for $m=m^{\prime }=0$, it is strictly positive. The sesqui-linearity of the complex scalar product follows from the fact that, according to (77) we have

$$\langle m_{1}+im_{2},i\left(m_{1}^{\prime }+im_{2}^{\prime }\right)\rangle =\langle m_{1}+im_{2},-m_{2}^{\prime }+im_{1}^{\prime }\rangle$$

$$=-{\langle m_{1}|m_{2}^{\prime }\rangle }_{H}+{\langle m_{2}|m_{1}^{\prime }\rangle }_{H}+i\left({\langle m_{1}|m_{1}^{\prime }\rangle }_{H}+{\langle m_{2}|m_{2}^{\prime }\rangle }_{H}\right)$$

which is the same result obtained by multiplying by *i* the right-hand side of (77).

(i) is clear from the definition (77).

(ii) follows from (77) by putting $m_{2}=m_{1}^{\prime }=0$.

To prove (iii), note that, since ${\left|A\right|}_{H}$ and $J_{H}$ commute, it follows that

$$\langle m_{1}+J_{H}m_{2}{,|A|}_{H}\left(m_{1}+J_{H}m_{2}\right)\rangle =\langle m_{1}+J_{H}m_{2}{,|A|}_{H}m_{1}+J_{H}{\left|A\right|}_{H}m_{2}\rangle$$

$$\overset{\left(77\right)}{=}\langle m_{1}{,|A|}_{H}m_{1}\rangle_{H}+\langle m_{2}{,|A|}_{H}m_{2}\rangle_{H}+i(\langle m_{1}{,|A|}_{H}m_{2}\rangle_{H}-\langle m_{2}{,|A|}_{H}m_{1}\rangle_{H})$$

But ${\left|A\right|}_{H}$ is hermitian for the (real valued) *H*–scalar product. Therefore,

$$\langle m_{1}{,|A|}_{H}m_{2}\rangle_{H}-\langle m_{2}{,|A|}_{H}m_{1}\rangle_{H}=\langle m_{1}{,|A|}_{H}m_{2}\rangle_{H}-{\langle |A|}_{H}m_{2},m_{1}\rangle_{H}$$

$$=\langle m_{1}{,|A|}_{H}m_{2}\rangle_{H}-\langle m_{1}{,|A|}_{H}m_{2}\rangle_{H}=0$$

It follows that ${\left|A\right|}_{H}$ is positive for the 〈·,·〉–scalar product because ${\left|A\right|}_{H}$ is positive for the *H*–scalar product and

$$\langle m_{1}+J_{H}m_{2}{,|A|}_{H}\left(m_{1}+J_{H}m_{2}\right)\rangle =\langle m_{1}{,|A|}_{H}m_{1}\rangle_{H}+\langle m_{2}{,|A|}_{H}m_{2}\rangle_{H}\geq 0$$

(iv) From the right-hand side of (77) it is clear that any orthogonal transformation of M gives rise to a unitary operator in $M\dot{+}J_{H}M$. In particular, the one-parameter group of orthogonal transformation of M

(80) $$V_{t}:=e^{tJ_{H}{\left|A\right|}_{H}}\equiv e^{itH}\;,\;t\in R$$

is unitary in the Hilbert space $\left(S_{C},\langle \;\cdot \;,\;\cdot \;\rangle \right)$. □

**Remark** **7.** *Note that, in (80), we have used for the H–complexified Hamiltonian evolution, acting on the complex Hilbert space $\left(S_{C},\langle \;\cdot \;,\;\cdot \;\rangle \right)$, the same notation $e^{itH}$ used in Appendices Appendix A.1 and Appendix A.2.*

### 4.5. The Standard Quantization of a Linear Classical Hamiltonian System

In Appendix A.2, we see that the solution of the equation of motion of a linear classical Hamiltonian system of n(∈N) degrees of freedom is obtained by reducing such a system to a sum of *n* independent one-dimensional harmonic oscillators.

For such systems, the naive quantization method described in Section 2.1 works perfectly, and the associated one-dimensional Schrödinger equation can be solved explicitly leading to the Hermite polynomials as eigenvectors of the quantum Hamiltonian and to the natural integers (after re-scaling of the masses and the frequencies) as eigenvalues. This leads to what in physics is called the *ground state representation of the Heisenberg commutation relations*, that is, a representation not on the space $L^{2}\left(R,dx\right)$, with Lebesgue measure, but on the space $L^{2}\left(R,\gamma \left(x\right)dx\right)$, where γ(x) is the standard Gaussian density. The advantage of this representation is that it continues to have a meaning when n=+∞, so that it is applicable also to (free) quantum fields.

Let us discuss this procedure more explicitly.

#### 4.5.1. The Quantized Linear Hamiltonian Evolutions

Remembering that, in the finite dimensional case, a linear Hamiltonian system on $M\dot{+}M$ is characterized by a real bi-linear Hamiltonian *H* with the associated quadratic form,

(81) $$H\left(q,p\right)\overset{\left(53\right)}{=}\frac{1}{2}{\langle p,p\rangle }_{T}+\frac{1}{2}{\langle q,Fq\rangle }_{T}$$

where *F* is a positive self-adjoint operator in the space $M_{T}=\left\{M,{\langle \cdot ,\cdot \rangle }_{T}\right\}$, one can choose a basis $\left(e_{j}\right)$ in $M_{T}$ such that

(82) $$H\left(q,p\right)=\frac{1}{2}\sum p_{j}^{2}+\frac{1}{2}\sum \omega_{j}^{2}q_{j}$$

where $q=\sum_{j}\;q_{j}e_{j};p=\sum p_{j}e_{j}$, and we suppose that all the $\omega_{j}^{2}$ are >0. Equation (82) is the Hamiltonian of a system of independent classical harmonic oscillators with frequencies $\omega_{j}$. The quantization of the *j*-th oscillator is performed, according to Schrödinger’s prescriptions (see Section 2.1), by the substitution

(83) $$\frac{1}{2}\left(p_{j}^{2}+\omega_{j}^{2}q_{j}^{2}\right)\to \frac{1}{2}\left(-\frac{\partial^{2}}{\partial q_{j}^{2}}+\omega_{j}^{2}q_{j}^{2}\right)=\frac{1}{2}H_{j}$$

If d:=dim(M)<+∞, the application of Schrödinger quantization leads to the operator

(84) $$H=\sum_{j}H_{j}\;=\sum_{j}\left(\frac{\partial^{2}}{\partial q_{j}^{2}}+\omega_{j}^{2}q_{j}^{2}\right)$$

and then to the dynamic group:

(85) $$e^{itH}=e^{itH_{1}}⊗\cdots ⊗e^{itH_{d}}$$

Defining, for each index *j*,

(86) $$\gamma_{j}\left(x\right)=\frac{e^{-\omega_{j}x^{2}/2}}{\sqrt{2\pi /\omega_{j}}}\;;\;\omega_{j}>0$$

one has

(87) $$\left(H_{j}-\omega_{j}\right)\gamma_{j}=0\Leftrightarrow e^{it(H_{j}-\omega_{j})}\gamma_{j}=\gamma_{j}$$

It is convenient, and always possible up to unitary isomorphism, to realize each evolution $e^{it(H_{j}-\omega_{j})}$ not on the space $L^{2}\left(R\right)$, but on $L^{2}\left(R,\gamma_{j}\left(x\right)dx\right)$.

In this realization, it takes the form $e^{itH_{\gamma_{j}}}$, where $H_{\gamma_{j}}$ has an ortho-normal basis of eigenvectors ${\left(h_{n}^{\left(j\right)}\right)}_{n}$, where $h_{n}^{\left(j\right)}$ is an appropriate $\omega_{j}$-dependent re-scaling of the *n*-th Hermite polynomial and

(88) $$h_{0}^{\left(j\right)}=1\;\left(:=\;\mathrm{constant}\;\mathrm{function}\;\mathrm{on}\;R\;\mathrm{equal}\;\mathrm{to}\;1\right)\;,\;\forall j\;,\;\forall n$$

The re-scalings are determined by the condition

(89) $$e^{itH_{\gamma_{j}}}h_{n}^{\left(j\right)}=e^{itn\omega_{j}}h_{n}^{\left(j\right)}\;,\;\forall n\in N\;,\;\forall j$$

With these notations, the global Schrödinger evolution is

(90) $$e^{itH_{\gamma }}=e^{itH_{\gamma_{1}}}⊗\cdots ⊗e^{itH_{\gamma_{j}}}⊗\cdots$$

and the set of its eigenvectors is given by the family

(91) $$\left\{\underset{j}{⨂}h_{n_{j}}^{\left(j\right)}:n_{j}=0\;\mathrm{for}\;\mathrm{almost}\;\mathrm{all}\;j\right\}$$

is an ortho-normal basis of

(92) $$\underset{j}{⨂}L^{2}\left(R,d\gamma_{j}\right)≅L^{2}\left(\prod_{j}R\;,\;\underset{j}{⨂}d\gamma_{j}\right)$$

which diagonalizes $H_{\gamma }$:

(93) $$e^{itH_{\gamma }}\left(\underset{j}{⨂}h_{n_{j}}^{\left(j\right)}\right)=e^{it\sum_{j}n_{j}\omega_{j}}\left(\underset{j}{⨂}h_{n_{j}}^{\left(j\right)}\right)$$

Everything stated in this section remains valid if tensor products are interpreted in the sense of incomplete von Neumann tensor products. For the reader’s convenience, this notion is briefly recalled in Appendix D.

#### 4.5.2. A Coordinate-Free Construction of the Ground State Representation

A coordinate-free way to construct the ground state representation is presented below.

One starts from a given linear Hamiltonian system with phase space

$\left(S,\omega \right)\equiv \left(M\dot{+}M,\omega \right)$ and quadratic Hamiltonian given by (see page. 15.)

H((q,p),(q′,p′)):=12〈p,p′〉T+12〈q,Fq′〉T=:qp,q′p′H∈R

We apply, to the real Hilbert space $\left(M,{\langle \;\cdot \;,\;\cdot \;\rangle }_{T}\right)$, the following result, due to M. Kac in finite dimensions and extended by I. Segal to infinite dimensions.

**Theorem** **8.** *There exists a stochastic process,*

(94) $$n:(M,{\langle \;\cdot \;,\;\cdot \;\rangle }_{T})\;⟼\;\mathrm{random}\;\mathrm{variables}\;\mathrm{in}\;a\;\mathrm{probability}\;\mathrm{space}$$

*unique up to stochastic equivalence, with the following properties:*

*(i)*  *n, defined by (94), is a real linear map.*
*(ii)*  *If $x_{n}\to x$ in M, then $n\left(x_{n}\right)\to n\left(x\right)$ in measure.*
*(iii)*  *For each $x_{1},\cdots ,x_{n}\in H$ and for each orthogonal transformation U:M→M the joint distributions of $n\left(x_{1}\right),\cdots ,n\left(x_{n}\right)$ and those of $n\left(Ux_{1}\right),\cdots ,n\left(Ux_{n}\right)$ coincide.*
*(iv)*  *If x,y,∈M and x⊥y then n(x) and n(y) are independent random variables.*

*The only processes with these properties are Gaussian processes indexed by M with zero mean and covariance given by the ${\langle \;\cdot \;,\;\cdot \;\rangle }_{T}$–product in M multiplied by a positive constant (which we will assume =1).*

**Definition** **5.** *The process defined by Theorem 8 is called the **unit Gaussian process over** $\left(M,{\langle \;\cdot \;,\;\cdot \;\rangle }_{T}\right)$, and one uses the notation*

$$L^{2}\left(M\right):=L_{C}^{2}\left(\Omega ,F,P\right)$$

*where (Ω,F,P) is any realization of the process (any two of these realizations being isomorphic as probability spaces).*

**Example** **1.**
$M=L^{2}\left(\left[0,1\right]\right)$

$$n:f\in L^{2}\left(\left[0,1\right]\right)\to n_{f}:=\int_{0}^{1}f\left(t\right)dw_{t}$$

*where the integral on the right-hand side is the Wiener–Ito stochastic integral with respect to the standard white noise $dw_{t}$ (increment process of the Brownian motion).*

**Remark** **8.** *If $\left(e_{J}\right)$ is any orthonormal basis of M, the family of independent Gaussian random variables $\left(n\left(e_{J}\right)\right)$ completely determines the unit Gaussian process n(·), so one can choose*

$$\left(\Omega ,F,P\right)=\prod_{dimM}\left(R,\gamma \left(x\right)dx\right)$$

*where*

$$\gamma \left(x\right)=\frac{e^{-x^{2}/2}}{\sqrt{2\pi }}$$

*is the standard Gaussian density, defining a probability measure on the Borel σ-algebra on R. In this case,*

(95) $$L^{2}\left(M\right)=L^{2}\left(\prod_{J}R,\prod_{J}\gamma \left(x\right)dx\right)≅\overset{\left(1\right)}{⨂}L^{2}\left(R,\gamma \left(x\right)dx\right)≅\overset{\left(\sqrt{\gamma }\right)}{⨂}L^{2}\left(R\right)$$

*where (1) (resp. $\left(\sqrt{\gamma }\right)$ denotes the family of (dimM)–functions on R identically equal to 1 (resp. $\sqrt{\gamma }$) and, for any family of Hilbert spaces $\left(H_{\alpha }\right)$ and of unit vectors $\varphi_{\alpha }\in H_{\alpha }$, ${⨂}^{\left(\varphi_{\alpha }\right)}H_{\alpha }$ denotes incomplete tensor product in the sense of von Neumann. In (95), the indices of all products are extended to a family of dimM)—factors that are all equal to each other.*

Finally, one uses the symplectic form ω to define a representation of the CCR on $L^{2}\left(M\right)$ and the field operators on which the classical Hamiltonian evolution, that preserves the symplectic form, acts as a one-parameter automorphism group on the Weyl algebra.

### 4.6. Exponential of a Hilbert Space

The material presented in this issue is taken from the work of H. Araki and J. Woods [38]. The notation Γ(H) is that of I.E. Segal, cf. e.g., [39].

Let H be a Hilbert space (real or complex). We will use the following notations interchangeably

$$\Gamma \left(H\right)\;,\;e^{H}\;,\;\exp\left(H\right)$$

to indicate the (infinite dimensional) Hilbert space,

(96) $$\overset{\infty }{\underset{n=0}{⨁}}{\left(\underset{sym}{⨂}H\right)}^{n}\;,\;{\left(\underset{sym}{⨂}H\right)}^{0}:=C\cdot \Phi$$

where Φ is a unit vector called *vacuum vector* and

$${\left(\underset{sym}{⨂}H\right)}^{n}$$

is the subspace of ${\left(⊗H\right)}^{n}=H⊗\cdots ⊗H$ (*n*–times) image of the projector,

(97) $$S:\varphi_{1}⊕\cdots ⊕\varphi_{n}\to \frac{1}{n!}\sum_{\pi \in S_{n}}\varphi_{\pi (1)}⊗\cdots ⊗\varphi_{\pi (n)}$$

where $S_{n}$ denotes the permutation group on {1,⋯,n}.

If $H=L^{2}\left(R\right){\left(⊗H\right)}^{n}≅L^{2}\left(R^{n}\right)$ and ${\left({⊗}_{sym}H\right)}^{n}$ is the space of square integrable symmetric functions of *n* real variables, the set of vectors

$$\varphi^{⊗n}:=\varphi ⊗\cdots ⊗\varphi \;\left(n--\mathrm{times}\right)$$

is total in ${\left({⊗}_{sym}H\right)}^{n}$ as deduced from the polarization identity

(98) $$\sum_{\pi \in S_{n}}\varphi_{\pi (1)}⊗\cdots ⊗\varphi_{\pi (n)}=\frac{1}{2^{n}}\sum_{\varepsilon_{1},\cdots ,\varepsilon_{n}\in \left\{\pm 1\right\}}\left(\prod_{j\in \{1,\ldots ,n\}}\varepsilon_{j}\right){\left(\sum \epsilon_{j}\varphi_{j}\right)}^{⊗n}$$

The notation exp(H) is justified by the following Lemma:

**Lemma** **3.** *(i) The vectors*

(99) $$exp\left(\varphi \right)=e^{\varphi }=\sum_{n=0}^{\infty }\frac{1}{\sqrt{n!}}{\left(⊗\varphi \right)}^{n}\;;\;\varphi \in H$$

*are a linearly independent total set in exp(H).*

*(ii)*

(100) $$\langle e^{\varphi },e^{\psi }\rangle =e^{\left(\varphi ,\psi \right)}\;;\;\varphi ,\psi \in H$$

*where 〈·,·〉 (resp. (·,·)) denotes the scalar product in (expH) (resp. H).*

*(iii) If $\left(H_{\alpha }\right)$ is a family of Hilbert spaces and ${\left({⨂}_{sym}H_{\alpha }\right)}^{0}:=C\cdot \Phi$ one has*

(101) $$\exp\left(\underset{\alpha }{⨁}H_{\alpha }\right)\equiv \overset{\left(\Phi \right)}{\underset{\alpha }{⨂}}\exp\left(H_{\alpha }\right)$$

*where the right-hand side of (101) is the **incomplete component**, in the sense of von Neumann, of the infinite tensor product ${⊗}_{sym}\exp\left(H_{\alpha }\right)$ relative to the family (*Φ*) (see formula (A11) for a more detailed description), and the identification is established by*

(102) $$\underset{\alpha }{⨂}e^{\psi_{\alpha }}⟼e^{\sum_{\alpha }\psi_{\alpha }}\;;\;\sum_{\alpha }\psi_{\alpha }\in {⊕}_{\alpha }H_{\alpha }$$

#### 4.6.1. The Boson Fock Functor

Let A:H→H be a linear contraction. From Lemma 3, it follows that the map

(103) $$e^{\varphi }\in \Gamma \left(H\right)\to e^{A\varphi }\in \Gamma \left(H\right)$$

has a unique linear extension

(104) Γ(A):Γ(H)→Γ(H)

and that

(105) Γ(A)isalinearcontraction

Moreover,

(106) $$\Gamma \left(a^{*}\right)=\Gamma {\left(A\right)}^{*}$$

(107) Γ(A·B)=Γ(A)·Γ(B);∥A∥,∥B∥≤1

Therefore, the operation Γ is a functor from the category of Hilbert spaces ≠{0} with linear contractions as morphisms to the category of infinite-dimensional Hilbert spaces with the same morphisms. One can prove [40] that, if *A* is not a linear contraction, Γ(A) cannot be a bounded operator.

(108) $$\Gamma \left(A\right)\cdot \Phi_{0}=\Phi_{0}\;\;(\Phi_{0}\;\mathrm{is}\;\mathrm{the}\;\mathrm{vacuum}\;\mathrm{vector})$$

(109) $$\Gamma \left(A\right){\left(\varphi ⊗\cdots ⊗\varphi \right)}_{n}=\left(A\varphi \right)⊗\cdots ⊗{\left(A\varphi \right)}_{n}$$

**Example** **2.** ***The number of particles operator***

*In the previous notations, let $U_{t}$(t∈R) be the one-parameter unitary group on H given by*

$$U_{t}:\varphi \in H\to \;e^{it}\varphi \in H$$

*Then $\Gamma (U_{t})$ is a strongly continuous one-parameter unitary group on Γ(H), therefore, by Stone’s theorem:*

$$\Gamma \left(e^{it}\right)=e^{itN}$$

*where N is a self-adjoint operator on Γ(H). On the other hand, by (109),*

$$\Gamma \left(e^{it}\right){\left(\varphi ⊗\cdots ⊗\varphi \right)}_{n}=e^{itn}{\left(\varphi ⊗\cdots ⊗\varphi \right)}_{n}$$

*then, for each n∈N,*

$$N{|}_{H^{{⊗}_{sym}n}}=n\cdot 1=\;\mathrm{multiplication}\;\mathrm{by}\;n$$

*If H is interpreted as the space of (quantum) states of a particle (e.g., a photon), Γ(H) will be the space of (quantum) states of a system with a variable (possibly infinite) number of particles of the same type (photons): the normalized vectors of the space (${⊗}_{sym}H^{n}$) correspond to those states of the system in which exactly n particles are present; transitions between states of these spaces relative to different values of n have nonzero probability in a relativistic context due to the equivalence between mass and energy and occur through processes of **creation** and **annihilation** of particles. The fact that, for each n, the space ${\left({⊗}_{sym}H\right)}^{n}$ is the eigenspace of the operator N corresponding to the eigenvalue n justifies the name **particle number operator** for N.*

#### 4.6.2. The Tensor Product Representation of Boson Fock Space

If H is any Hilbert space and $\left(e_{\alpha }\right)$ is an orthonormal basis in H, we have

$$H≅\underset{\alpha }{⨁}\left(C\cdot e_{\alpha }\right)$$

so,

(110) $$e^{H}\;\overset{\left(101\right)}{≅}\;\overset{\left(1_{\alpha }\right)}{\underset{\alpha }{⨂}}e^{C\cdot e_{\alpha }}$$

Therefore (see (95)), computations on exp(H) are reduced to computations on exp(C). On the other hand,

$${\left(⊗C\right)}^{n}\equiv {\left(\underset{sym}{⨂}C\right)}^{n}\equiv C$$

so that

(111) $$exp\left(C\right)\overset{\left(96\right)}{=}\overset{\infty }{\underset{n=0}{⨁}}C\cdot \Phi_{n}$$

where we have introduced the symbol $\Phi_{n}$ to label the vacuum states of each term of the direct sum in (111).

To summarize, the space expH is isomorphic to an infinite tensor product over a family of unit vectors of a set of complex separable Hilbert spaces.

The space expH, introduced by the Soviet physicist V. Fock [41] for the description of quantum systems of infinitely many particles, is called the **Fock space** of H. The first rigorous definition of this space, and analysis of the important operators that operate on it, is due to J. M. Cook [42].

### 4.7. Boson Fock Second Quantization of the Complexified Unitary Dynamics

We can now use the results of Section 4.6.1 to apply directly the boson Fock functor to the complexified unitary Hamiltonian evolution (80) described in Section 4.4.1 and Section 4.4.2.

Remember that this evolution has the form $\left(V_{t}\right)=e^{itH}$ and acts on the complex Hilbert space $\left(S_{C},\langle \;\cdot \;,\;\cdot \;\rangle \right)$ (see Theorem 7). So, its Fock second quantization, as described in Section 4.6.1, always exists and give a one-parameter unitary group on the Fock space. This unitary group has a particularly simple form if the Hamiltonian satisfies the conditions introduced in Appendix A.2, namely,

$$S_{C}=M⊕J_{H}M≅\underset{j}{⨁}Ce_{j}$$

where

$$\left(e_{j}\right)=\;\;\mathrm{real}\;o.n.\;\mathrm{basis}\;\mathrm{of}\;M=\;\mathrm{complex}\;o.n.\;\mathrm{basis}\;\mathrm{of}\;M⊕J_{H}M$$

and that the Hamiltonian evolution can be represented in the diagonal form

$$V_{t}≅\underset{j}{⨁}e^{it\omega_{j}}e_{j}e_{j}^{*}$$

acting on $S_{C}$ and, up to an additive constant in the Hamiltonian, one can suppose that j∈{0,1,…,n} and that $\omega_{0}=1$. Therefore the boson Fock second quantization of this evolution will act on

(112) $$\Gamma \left(S_{C}\right)≅\overset{\left(\Phi_{0;j}\right)}{⨂}\Gamma \left(C\cdot e_{j}\right)$$

where, for each *j*, $\Phi_{0;j}$ denotes the vacuum vector of $\Gamma (C\cdot e_{j})$, and its action is given by

(113) $$\Gamma \left(V_{t}\right)≅\underset{j}{⨂}\Gamma \left(e^{it\omega_{j}}\right)=e^{itd\Gamma (H)}$$

where the last equality is the definition of the self-adjoint operator dΓ(H) on $\Gamma (S_{C})$, the infinitesimal generator of the second quantized one-parameter unitary group $\Gamma (V_{t})$. Note that, by (108), $\Gamma (V_{t})$, the right-hand side of (113) is well defined on a dense set of vectors of $\Gamma (S_{C})$, and, therefore, by unitarity, on the whole of $\Gamma (S_{C})$.

If we denote $\left(\Phi_{j;n}\right)$ the monic orthogonal basis in $\Gamma (C\cdot e_{j})$, the family

$$\left\{\underset{j}{⨂}\Phi_{j;n_{j}}:n_{j}=0\;,\;\mathrm{for}\;\mathrm{almost}\;\mathrm{all}\;j\right\}$$

is an ortho-normal basis of $\Gamma (S_{C})$ that diagonalizes dΓ(H).

More explicitly,

$$e^{itd\Gamma (H)}\left(\underset{j}{⨂}\Phi_{j;n_{j}}\right)=e^{it\sum n_{j}\omega_{j}}\left(\underset{j}{⨂}\Phi_{j;n_{j}}\right)$$

But then the map

$$\underset{j}{⨂}\Phi_{j;n_{j}}\to ⨂h_{n_{j}}^{\left(j\right)}$$

extends to a unitary isomorphism

$$S:\Gamma \left(S_{C}\right)\to L_{C}^{2}\left(M\right)$$

with the property

$$S^{*}\cdot e^{itH_{\gamma }}\cdot S=e^{itd\Gamma (H)}$$

That is, the dynamics obtained by applying the Schrödinger quantization prescriptions (suitably renormalized if dim(M)=+∞) is unitarily equivalent to the dynamics obtained by performing the following operations:

(i) We start from the classical Hamiltonian dynamics $\left(V_{t}\right)$ on the phase space $M⊗M^{*}$.

(ii) We apply the complexification operation defined by the Hamiltonian, obtaining the complex Hilbert space $S_{C}$ and the one-parameter unitary group $\left(V_{t}\right)$.

(iii) We take the “exponential” of the result (functor Γ): $\Gamma \left(S_{C}\right)),\Gamma \left(V_{t}\right))$.

## 5. Conclusions

In this work, we show that every linear Hamiltonian system admits a canonical, intrinsic, and unambiguous quantization, and that this quantization arises directly from the geometric structure of the classical dynamics. The procedure is coordinate-independent, conceptually transparent, and naturally extendable to infinite dimensional cases (free fields). The explicit form of this extension has been illustrated in Section 4.7 in the case when the complexified Hamiltonian has a pure-point spectrum. In this case, the intrinsic quantization obtained from the classical dynamics is unitarily equivalent to the standard (Schrödinger/Heisenberg) quantization of the system of harmonic oscillators into which the linear system decomposes.

The result can be interpreted as a reconciliation between the classical and quantum formalisms, demonstrating that, at least for linear systems, the emergence of a complex structure on the phase space under which the Hamiltonian evolution becomes unitary is not an external imposition, but a process already inscribed within the very structure of Hamiltonian dynamics.

This purely mathematical reconciliation motivated the initiators of this line of research, essentially Segal and then Mackey.

For non-linear Hamiltonian systems, such a complete picture is not available, even if some partial results exist, like those mentioned in the Abstract or the identification of *wave functions* with *complex square roots of measures* [43].

However this is a result only on the mathematical structure of quantum theory, which gives no information on some fundamental aspects of such theory such as, for example, Planck’s constant: no physical scale enters this mathematical structure. Note that this holds also for classical Hamiltonian dynamics where masses, the gravitational constant, charges, etc., are not deduced, but placed from the outside. 

## Data Availability

The original contributions presented in this study are included in the article. Further inquiries can be directed to the corresponding author.

## Appendix A. Solutions of the Equations of Motion

This section is not essential and can be skipped.

With the notation (49), the equations of motion (42) become

(A1) $$\dot{q_{t}}=\partial_{p}H=p_{t}\;;\;\dot{p_{t}}=-\partial_{q}H=-Fq_{t}$$

⇔∂tqtpt=pt−Fqt=01−10Fqtpt=01−10F001qtpt

⇔∂tqtpt=01−F0qtpt=:Aqtpt

with initial condition

q0p0

The operator

(A2) $$A:=\left(\begin{array}{cc} 0 & 1\\ -F & 0 \end{array}\right)$$

can be explicitly exponentiated using the exponential series because

$$A^{2}={\left(\begin{array}{cc} 0 & 1\\ -F & 0 \end{array}\right)}^{2}=\left(\begin{array}{cc} 0 & 1\\ -F & 0 \end{array}\right)\left(\begin{array}{cc} 0 & 1\\ -F & 0 \end{array}\right)=\left(\begin{array}{cc} -F & 0\\ 0 & -F \end{array}\right)=\left(-1\right)\left(\begin{array}{cc} F & 0\\ 0 & F \end{array}\right)$$

which implies

$$A^{2n}={\left(-1\right)}^{n}\left(\begin{array}{cc} F^{n} & 0\\ 0 & F^{n} \end{array}\right)={\left(-1\right)}^{n}{\left(\begin{array}{cc} F & 0\\ 0 & F \end{array}\right)}^{n}$$

$$A^{2n+1}={\left(-1\right)}^{n}\left(\begin{array}{cc} F^{n} & 0\\ 0 & F^{n} \end{array}\right)\left(\begin{array}{cc} 0 & 1\\ -F & 0 \end{array}\right)={\left(-1\right)}^{n}{\left(\begin{array}{cc} F & 0\\ 0 & F \end{array}\right)}^{n}\left(\begin{array}{cc} 0 & 1\\ -F & 0 \end{array}\right)$$

It follows that

$$e^{tA}=\sum_{n=0}^{+\infty }\frac{t^{n}}{n!}A^{n}=\sum_{p=0}^{+\infty }\frac{t^{2p}}{(2p)!}A^{2p}+\sum_{p=0}^{+\infty }\frac{t^{2p+1}}{(2p+1)!}A^{2p+1}$$

$$=\sum_{p=0}^{+\infty }\frac{t^{2p}}{(2p)!}{\left(-1\right)}^{p}\left(\begin{array}{cc} F^{p} & 0\\ 0 & F^{p} \end{array}\right)+\sum_{p=0}^{+\infty }\frac{t^{2p+1}}{(2p+1)!}{\left(-1\right)}^{p}\left(\begin{array}{cc} F^{p} & 0\\ 0 & F^{p} \end{array}\right)\left(\begin{array}{cc} 0 & 1\\ -F & 0 \end{array}\right)$$

Using

$$F^{p}={\left(F^{1/2}\right)}^{2p}={\left(F^{1/2}\right)}^{2p+1}F^{-1/2}$$

we find

$$e^{tA}=\sum_{p=0}^{+\infty }\frac{t^{2p}}{(2p)!}{\left(-1\right)}^{p}\left(\begin{array}{cc} {\left(F^{1/2}\right)}^{2p} & 0\\ 0 & {\left(F^{1/2}\right)}^{2p} \end{array}\right)$$

$$+\sum_{p=0}^{+\infty }\frac{t^{2p+1}}{(2p+1)!}{\left(-1\right)}^{p}\left(\begin{array}{cc} {\left(F^{1/2}\right)}^{2p+1}F^{-1/2} & 0\\ 0 & {\left(F^{1/2}\right)}^{2p+1}F^{-1/2} \end{array}\right)\left(\begin{array}{cc} 0 & 1\\ -F & 0 \end{array}\right)$$

$$=\left(\begin{array}{cc} \sum_{p=0}^{+\infty }{\left(-1\right)}^{p}\frac{1}{(2p)!}{\left(tF^{1/2}\right)}^{2p} & 0\\ 0 & \sum_{p=0}^{+\infty }{\left(-1\right)}^{p}\frac{1}{(2p)!}{\left(tF^{1/2}\right)}^{2p} \end{array}\right)$$

$$+\sum_{p=0}^{+\infty }{\left(-1\right)}^{p}\frac{1}{(2p+1)!}\left(\begin{array}{cc} {\left(tF^{1/2}\right)}^{2p+1} & 0\\ 0 & {\left(tF^{1/2}\right)}^{2p+1} \end{array}\right)\left(\begin{array}{cc} F^{-1/2} & 0\\ 0 & F^{-1/2} \end{array}\right)\left(\begin{array}{cc} 0 & 1\\ -F & 0 \end{array}\right)$$

$$=\left(\begin{array}{cc} \cos(tF^{1/2}) & 0\\ 0 & \cos(tF^{1/2}) \end{array}\right)$$

$$+\left(\begin{array}{cc} \sum_{p=0}^{+\infty }{\left(-1\right)}^{p}\frac{1}{(2p+1)!}{\left(tF^{1/2}\right)}^{2p+1} & 0\\ 0 & \sum_{p=0}^{+\infty }{\left(-1\right)}^{p}\frac{1}{(2p+1)!}{\left(tF^{1/2}\right)}^{2p+1} \end{array}\right)\left(\begin{array}{cc} 0 & F^{-1/2}\\ -F^{1/2} & 0 \end{array}\right)$$

$$=\left(\begin{array}{cc} \cos(tF^{1/2}) & 0\\ 0 & \cos(tF^{1/2}) \end{array}\right)+\left(\begin{array}{cc} \sin(tF^{1/2}) & 0\\ 0 & \sin(tF^{1/2}) \end{array}\right)\left(\begin{array}{cc} 0 & F^{-1/2}\\ -F^{1/2} & 0 \end{array}\right)$$

$$=\left(\begin{array}{cc} \cos(tF^{1/2}) & 0\\ 0 & \cos(tF^{1/2}) \end{array}\right)+\left(\begin{array}{cc} 0 & F^{-1/2}\sin\left(tF^{1/2}\right)\\ -F^{1/2}\sin\left(tF^{1/2}\right) & 0 \end{array}\right)$$

where in the last identity we have used the fact that functions of the same self–adjoint operator commute. In conclusion,

(A3) $$e^{tA}=\left(\begin{array}{cc} \cos(tF^{1/2}) & F^{-1/2}\sin\left(tF^{1/2}\right)\\ -F^{1/2}\sin\left(tF^{1/2}\right) & \cos(tF^{1/2}) \end{array}\right)$$

### Appendix A.1. Example 1. *1*–Degree of Freedom

Choosing M=R; $M^{*}≅R$ and fixing the mass of the oscillator to be 1, we have

(A4) $$H\left(q,p\right)=\frac{1}{2}p^{2}+\frac{1}{2}\omega^{2}q^{2}\;;\;p,q\in R$$

where $\omega^{2}\left(=F\right)$ is a positive real number. From the equations of motion:

$$\frac{d}{dt}\left(\begin{array}{c} q\\ p \end{array}\right)=\left(\begin{array}{c} p\\ -Fq \end{array}\right)=\left(\begin{array}{c} p\\ -\omega^{2}q \end{array}\right)$$

it follows that, in the notations introduced above:

$$H≅R^{2}\;;\;{\langle \left(\begin{array}{c} q\\ p, \end{array}\right)\left(\begin{array}{c} q^{\prime }\\ p^{\prime } \end{array}\right)\rangle }_{H}=\frac{1}{2}pp^{\prime }+\frac{1}{2}\omega^{2}qq^{\prime }$$

The linear Hamiltonian evolution is given by

$$V_{t}=e^{tA}$$

and its infinitesimal generator is

(A5) $$A\left(\begin{array}{c} q\\ p \end{array}\right)=\left(\begin{array}{c} p\\ -\omega^{2}q \end{array}\right)$$

Consequently its polar decomposition is given by

$$\left|A\right|\left(\begin{array}{c} q\\ p \end{array}\right)=\left(\begin{array}{c} \omega q\\ \omega p \end{array}\right)\;;\;J\left(\begin{array}{c} p\\ q \end{array}\right)=\left(\begin{array}{c} \omega^{-1}p\\ \omega^{-1}q\; \end{array}\right)$$

The explicit calculation of the linear Hamiltonian evolution identified to its complexified unitary version is given by

$$e^{tJ|A|}\left(\begin{array}{c} q\\ p \end{array}\right)\equiv e^{itH}\left(\begin{array}{c} q\\ p \end{array}\right)=$$

$$=\sum_{n}\frac{t^{n}}{n!}J^{n}{\left|A\right|}^{n}\left(\begin{array}{c} q\\ p \end{array}\right)=\;(\sin\mathrm{ce}\;J|A|=\left|A|J\right)$$

$$=\sum_{n}\frac{t^{2n}}{(2n)!}{\left(-1\right)}^{n}{\left|A\right|}^{2n}\left(\begin{array}{c} q\\ p \end{array}\right)+\sum_{n}\frac{t^{2n+1}}{(2n+1)!}{\left(-1\right)}^{n}J{\left|A\right|}^{2n+1}\left(\begin{array}{c} q\\ p \end{array}\right)\;\left(\sin\mathrm{ce}\;J^{2}=-1\right)$$

$$=\sum_{n}\frac{t^{2n}}{(2n)!}{\left(-1\right)}^{n}\omega^{2n}\left(\begin{array}{c} q\\ p\\ +J \end{array}\right)\sum_{n}\frac{t^{2n+1}}{(2n+1)!}{\left(-1\right)}^{n}\omega^{2n+1}\left(\begin{array}{c} q\\ p \end{array}\right)=$$

$$=\left(cos\omega t\right)\left(\begin{array}{c} p\\ q \end{array}\right)+J\left(sin\omega t\right)\left(\begin{array}{c} q\\ p \end{array}\right)\equiv e^{i\omega t}\left(\begin{array}{c} q\\ p \end{array}\right)$$

where the symbol ≡ means that we use the standard notations for the complex structure on $R^{2}$.

This example shows, in the simplest case of 1 degree of freedom, how a linear Hamiltonian dynamics, driven by an Hamiltonian *H*, on the classical phase space $R^{2}$ is equivalent to a unitary dynamics on the *H*-complexification of the phase space, which is isomorphic to C. In the next section, we extend this example to the case of *n* degrees of freedom.

### Appendix A.2. Example 2. Arbitrary Number of Degrees of Freedom

We write the Hamiltonian in diagonal form:

(A6) $$H\left(q,p\right)=\frac{1}{2}\sum_{j}p_{j}^{2}+\frac{1}{2}\sum_{j}\omega_{j}^{2}q_{j}^{2}$$

where the $\omega_{j}^{2}$ are the eigenvalues of *F*. So, in this case,

$$H=\underset{j}{⨁}H_{j}\;;\;H_{j}≅R^{2}$$

$${\langle \left(\begin{array}{c} q\\ p \end{array}\right)\left(\begin{array}{c} q^{\prime }\\ p^{\prime } \end{array}\right)\rangle }_{H_{j}}=\frac{1}{2}pp^{\prime }+\frac{1}{2}\omega_{j}^{2}qq^{\prime }$$

The linear Hamiltonian evolution is given by

$$e^{tA}=\underset{j}{⨁}e^{tA_{j}}$$

where each $A_{j}$ is the $\omega_{j}$–analogue of (A5):

(A7) $$A_{j}:\left(\begin{array}{c} q\\ p \end{array}\right)\in H_{j}\to \left(\begin{array}{c} p\\ -\omega_{j}^{2}q \end{array}\right)$$

hence the unitary and the modulus in the polar decomposition of *A* are given respectively by

$$J=\underset{j}{⨁}J_{j}\;;\;|A|=\underset{j}{⨁}\left|A_{j}\right|$$

The evolution in the complexified Hilbert space is given by

$$e^{itH}:\underset{j}{⨁}\left(\begin{array}{c} q_{j}\\ p_{j} \end{array}\right)\to \underset{j}{⨁}e^{it\omega_{j}}\left(\begin{array}{c} q_{j}\\ p_{j} \end{array}\right)$$

So, the case of many degrees of freedom is reduced to a direct sum of cases with 1–degree of freedom.

## Appendix B. Proof of Proposition 1

**Proof.** From (57) and using the fact that *F* (hence also $F^{-1}$) is *T*–hermitian, one deduces that

X+qp,qpH=qp,XqpH=qp,F001Xq′p′T+˙T

=qp,F001XF001−1F001q′p′T+˙T

=qp,F−1001XF001F001q′p′T+˙T

=F001XF−1001*qp,F001q′p′T+˙T

=F−1001*X*F001*qp,F001q′p′T+˙T

=(F−1)*001X*F*001qp,F001q′p′T+˙T

=F−1001X*F001qp,q′p′H,∀qp,q′p′∈M+˙M

So, the relation between the two adjoints of *X* is (59). Since *A* is given by (56), one has

(A8) $$A^{*}={\left(\begin{array}{cc} 0 & 1\\ -F & 0 \end{array}\right)}^{*}=\left(\begin{array}{cc} 0 & -F\\ 1 & 0 \end{array}\right)$$

In fact:

qp,Aq′p′T+˙T=qp,01−F0q′p′T+˙T=qp,p′−Fq′T+˙T

$$=\frac{1}{2}{\langle q,p^{\prime }\rangle }_{T}+\frac{1}{2}{\langle p,-Fq^{\prime }\rangle }_{T}=\frac{1}{2}{\langle q,p^{\prime }\rangle }_{T}+\frac{1}{2}{\langle -Fp,q^{\prime }\rangle }_{T}=\frac{1}{2}{\langle -Fp,q^{\prime }\rangle }_{T}+\frac{1}{2}{\langle q,p^{\prime }\rangle }_{T}$$

=−Fpq,q′p′T+˙T=0−F10qp,q′p′T+˙T=A*qp,q′p′T+˙T

It follows that

(A9) $$A^{*}A=\left(\begin{array}{cc} 0 & -F\\ 1 & 0 \end{array}\right)\left(\begin{array}{cc} 0 & 1\\ -F & 0 \end{array}\right)=\left(\begin{array}{cc} F^{2} & 0\\ 0 & 1 \end{array}\right)$$

Therefore (60) holds. Since

$$\left(\begin{array}{cc} 0 & 1\\ -1 & 0 \end{array}\right){\left|A\right|}_{T}=\left(\begin{array}{cc} 0 & 1\\ -1 & 0 \end{array}\right)\left(\begin{array}{cc} F & 0\\ 0 & 1 \end{array}\right)=\left(\begin{array}{cc} 0 & 1\\ -F & 0 \end{array}\right)=A$$

the uniqueness of the polar decomposition implies that, with the notation (61), (62) holds. From this we deduce that the *H*–adjoint of *A* is

$$A^{+}\overset{\left(59\right)}{=}\left(\begin{array}{cc} F^{-1} & 0\\ 0 & 1 \end{array}\right)A^{*}\left(\begin{array}{cc} F & 0\\ 0 & 1 \end{array}\right)\overset{\left(A8\right)}{=}\left(\begin{array}{cc} F^{-1} & 0\\ 0 & 1 \end{array}\right)\left(\begin{array}{cc} 0 & -F\\ 1 & 0 \end{array}\right)\left(\begin{array}{cc} F & 0\\ 0 & 1 \end{array}\right)$$

$$=\left(\begin{array}{cc} 0 & -1\\ 1 & 0 \end{array}\right)\left(\begin{array}{cc} F & 0\\ 0 & 1 \end{array}\right)=\left(\begin{array}{cc} 0 & -1\\ F & 0 \end{array}\right)=-A$$

which is (63). Therefore,

$$A^{+}A\overset{\left(56\right)}{=}\left(\begin{array}{cc} 0 & -1\\ F & 0 \end{array}\right)\left(\begin{array}{cc} 0 & 1\\ -F & 0 \end{array}\right)=\left(\begin{array}{cc} F & 0\\ 0 & F \end{array}\right)$$

so that (64) holds. Hence, the polar decomposition of *A* in the *H*–space is given by the equation

$$A=J_{H}{\left|A\right|}_{H}\overset{\left(64\right)}{\Leftrightarrow }\left(\begin{array}{cc} 0 & 1\\ -F & 0 \end{array}\right)=J_{H}\left(\begin{array}{cc} F^{1/2} & 0\\ 0 & F^{1/2} \end{array}\right)$$

$$\Leftrightarrow J_{H}=\left(\begin{array}{cc} 0 & 1\\ -F & 0 \end{array}\right)\left(\begin{array}{cc} F^{-1/2} & 0\\ 0 & F^{-1/2} \end{array}\right)=\left(\begin{array}{cc} 0 & F^{-1/2}\\ -F^{1/2} & 0 \end{array}\right)$$

which is (65). In conclusion, the polar decomposition of *A* in the *H*–space is given by (66). The *H*–adjoint of $J_{H}$ is given by

$$J_{H}^{+}\overset{\left(59\right)}{=}\left(\begin{array}{cc} F^{-1} & 0\\ 0 & 1 \end{array}\right)J_{H}^{*}\left(\begin{array}{cc} F & 0\\ 0 & 1 \end{array}\right)\overset{\left(65\right)}{=}\left(\begin{array}{cc} F^{-1} & 0\\ 0 & 1 \end{array}\right){\left(\begin{array}{cc} 0 & F^{-1/2}\\ -F^{1/2} & 0 \end{array}\right)}^{*}\left(\begin{array}{cc} F & 0\\ 0 & 1 \end{array}\right)$$

$$=\left(\begin{array}{cc} F^{-1} & 0\\ 0 & 1 \end{array}\right){\left(\begin{array}{cc} 0 & F^{-1/2}\\ -F^{1/2} & 0 \end{array}\right)}^{*}\left(\begin{array}{cc} F & 0\\ 0 & 1 \end{array}\right)$$

$$=\left(\begin{array}{cc} F^{-1} & 0\\ 0 & 1 \end{array}\right)\left(\begin{array}{cc} 0 & -F^{1/2}\\ F^{-1/2} & 0 \end{array}\right)\left(\begin{array}{cc} F & 0\\ 0 & 1 \end{array}\right)$$

$$=\left(\begin{array}{cc} 0 & -F^{-1/2}\\ F^{-1/2} & 0 \end{array}\right)\left(\begin{array}{cc} F & 0\\ 0 & 1 \end{array}\right)=\left(\begin{array}{cc} 0 & -F^{-1/2}\\ F^{1/2} & 0 \end{array}\right)\overset{\left(65\right)}{=}-J_{H}$$

which is (67). We know that $J_{H}$ is a unitary in the real Hilbert space *H*, i.e., an orthogonal transformation. So

$$J_{H}^{+}J_{H}=J_{H}J_{H}^{+}=1$$

Since $J_{H}^{+}J_{H}=J_{H}J_{H}^{+}=-J_{H}^{2}$, this is confirmed by the direct calculation:

$$J_{H}^{2}=\left(\begin{array}{cc} 0 & F^{-1/2}\\ -F^{1/2} & 0 \end{array}\right)\left(\begin{array}{cc} 0 & F^{-1/2}\\ -F^{1/2} & 0 \end{array}\right)=\left(\begin{array}{cc} -1 & 0\\ 0 & -1 \end{array}\right)=-\left(\begin{array}{cc} 1 & 0\\ 0 & 1 \end{array}\right)$$

which is (68). □

## Appendix C. Proof of Theorem 6

**Proof.** 

$$J_{H}X=\left(\begin{array}{cc} 0 & F^{-1/2}\\ -F^{1/2} & 0 \end{array}\right)\left(\begin{array}{cc} B & C\\ D & E \end{array}\right)=\left(\begin{array}{cc} F^{-1/2}D & F^{-1/2}E\\ -F^{1/2}B & -F^{1/2}C \end{array}\right)$$

$$XJ_{H}=\left(\begin{array}{cc} B & C\\ D & E \end{array}\right)\left(\begin{array}{cc} 0 & F^{-1/2}\\ -F^{1/2} & 0 \end{array}\right)=\left(\begin{array}{cc} -CF^{1/2} & BF^{-1/2}\\ -EF^{1/2} & DF^{-1/2} \end{array}\right)$$

So,

$$F^{-1/2}D=-CF^{1/2}\Leftrightarrow D=-F^{1/2}CF^{1/2}$$

$$F^{-1/2}E=BF^{-1/2}\Leftrightarrow E=F^{1/2}BF^{-1/2}$$

$$-F^{1/2}B=-EF^{1/2}\Leftrightarrow F^{1/2}BF^{-1/2}=E$$

$$-F^{1/2}C=DF^{-1/2}\Leftrightarrow -F^{1/2}CF^{1/2}=D$$

$$XJ_{H}=\left(\begin{array}{cc} B & C\\ -F^{1/2}CF^{1/2} & F^{1/2}BF^{-1/2} \end{array}\right)\left(\begin{array}{cc} 0 & F^{-1/2}\\ -F^{1/2} & 0 \end{array}\right)$$

$$=\left(\begin{array}{cc} -CF^{1/2} & BF^{-1/2}\\ -F^{1/2}B & -F^{1/2}C \end{array}\right)$$

So,

$$X=\left(\begin{array}{cc} B & C\\ -F^{1/2}CF^{1/2} & F^{1/2}BF^{-1/2} \end{array}\right)$$

Verification:

$$J_{H}X=\left(\begin{array}{cc} 0 & F^{-1/2}\\ -F^{1/2} & 0 \end{array}\right)\left(\begin{array}{cc} B & C\\ -F^{1/2}CF^{1/2} & F^{1/2}BF^{-1/2} \end{array}\right)=\left(\begin{array}{cc} CF^{1/2} & BF^{-1/2}\\ -F^{1/2}B & -F^{1/2}C \end{array}\right)$$

□

## Appendix D. A Short Digression on Infinite Tensor Products of Hilbert Spaces

The advantage of the representation described by (91), (92), and (93) is that it also makes sense in the infinite dimensional case, provided the Hilbert spaces tensor products in these identities are replaced by **incomplete tensor products**.

This notion was introduced by J. von Neumann in [44] (see [45] vol.4 ex. 11.5.19 for a more updated exposition). Heuristically, if *T* is an infinite set, the symbol

(A10) $$\overset{\left(\varphi_{\alpha }\right)}{\underset{\alpha \in T}{⨂}}K_{\alpha }\;;\;\varphi_{\alpha }\in K_{\alpha }\;;\;∥\varphi_{\alpha }∥=1$$

denotes the completion of the vector space, generated by the formal infinite products

(A11) $$\underset{\alpha \in T}{⨂}\psi_{\alpha }\;;\;\psi_{\alpha }\in K_{\alpha }\;;\;\psi_{\alpha }=\varphi_{\alpha }\;\;\mathrm{for}\;\mathrm{almost}\;\mathrm{all}\;\alpha \;$$

(with the natural relations due to the multi-linearity of the tensor product) and for the scalar product:

(A12) $$\langle \underset{\alpha }{⨂}\psi_{\alpha },\underset{\alpha }{⨂}\psi_{\alpha }^{\prime }\rangle =\prod_{\alpha \in T}\langle \psi_{\alpha },\psi_{\alpha }^{\prime }\rangle$$

Note that the right-hand side of (A12) makes sense because for almost all α one has

$$\langle \psi_{\alpha },\psi_{\alpha }^{\prime }\rangle ={∥\varphi_{\alpha }∥}^{2}\overset{\left(A10\right)}{=}1$$

In the case considered in Section 4.5.1, it is sufficient to replace the space (92) by

(A13) $$\overset{\left(1\right)}{\underset{j}{⨂}}L^{2}\left(R,d\gamma_{j}\right)≅L^{2}\left(\prod_{j}R\;,\;\underset{j}{⨂}d\gamma_{j}\right)$$

where ${⨂}_{j}^{\left(1\right)}$ denotes the incomplete tensor product with respect to the family $1_{j}:=1$ (for all *j*), where the function 1 is defined by (88), so that the family (91) is an ortho-normal basis of ${⨂}_{j}^{\left(1\right)}L^{2}\left(R,d\gamma_{j}\right)$ and the right hand side of (A13) makes sense for any index set because the $d\gamma_{j}$ are probability measures.

Finally, for n=0 (89) gives

(A14) $$e^{itH_{\gamma_{j}}}h_{0}^{\left(j\right)}=e^{itH_{\gamma_{j}}}1=1\;,\;\forall j$$

and therefore the identity

(A15) $$e^{itH_{\gamma }}\left(\overset{\left(1\right)}{\underset{j}{⨂}}h_{n_{j}}^{\left(j\right)}\right)=e^{it\sum_{j}n_{j}\omega_{j}}\left(\overset{\left(1\right)}{\underset{j}{⨂}}h_{n_{j}}^{\left(j\right)}\right)$$

replacing (93), makes sense because on the right hand side almost all $n_{j}=0$.

So, $e^{itH_{\gamma }}$ is a one-parameter unitary group on ${⨂}^{\left(1\right)}L^{2}\left(R\right)$.
